# Organosolv Lignin-Based Electrospun Nanofibers: Stable Compositions and Morphological Insights

**DOI:** 10.1021/acssuschemeng.5c09295

**Published:** 2026-01-15

**Authors:** Paula Martínez Cánovas, Salvatore Cito, Francisco Medina, Joan Rosell-Llompart

**Affiliations:** † Department of Chemical Engineering, 16777 Universitat Rovira i Virgili, Tarragona E-43007, Spain; ‡ Department of Mechanical Engineering, 204165 Universitat Rovira i Virgili, Tarragona E-43007, Spain; § Catalan Institution for Research and Advanced Studies - ICREA, Barcelona E-08010, Spain

**Keywords:** electrospinning, organosolv lignin, nanofiber, hollow fiber, hardwood, softwood, eco-friendly, rheology

## Abstract

Lignin, a major component of lignocellulosic biomass, has been gaining interest as a sustainable alternative to petroleum-based polymers (e.g., polyacrylonitrile, PAN), and its valorization has included the production of nanofibers by electrospinning. In this work, we identify stable compositions leading to robust electrospinning from two different sources of organosolv lignin (OL), softwood (SOL), and hardwood (HOL), combined with variable concentrations of poly­(ethylene oxide) (PEO) of different molecular weights. Robust spinning of the solutions is sought while minimizing the required binder polymer and maximizing the lignin content in the produced fibers. The stability of the electrospinning process was rigorously monitored by imaging the Taylor cone (TC) during fiber formation by high-speed video, while solution rheology was analyzed to characterize the solutions and aid in understanding the fiber formation mechanism. The internal structure of the fibers was characterized by focused ion beam field emission scanning electron microscopy (FIB-FESEM), revealing the presence of internal voids in all compositions. When lignin was predominant, the fiber diameter was systematically smaller for HOL than for SOL, possibly due to the smaller molecular weight, revealing submicrometer fiber diameters in both cases. Finally, on the basis of these findings, a fiber formation mechanism is proposed.

## Graphical abstract

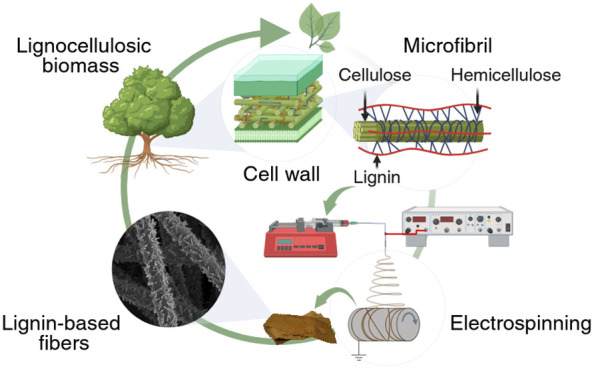

## Introduction

Lignin is gaining attention as a low-cost renewable resource and sustainable alternative to petroleum-based materials, mainly as a carbon source.^1–3^ Lignin extraction methods from lignocellulosic biomass influence the chemical properties, and thus the potential applications, of the material obtained. Among the best known methods for the extraction of lignin,^4^ namely kraft, organosolv, and lignosulfonate, the organosolv method stands out due to cleaner fractionation leading to higher quality of the extracted lignin.^5^ Therefore, efforts are underway to develop organosolv-lignin-based structures for various applications, particularly in fiber form for nanotechnology.^6^

Among other fiber production methods, electrospinning is uniquely able to generate uniform nanofibers.^7–10^ In previous studies, electrospun fibers have been produced from kraft,^11–15^ lignosulfonate,^16–18^ and organosolv lignin.^19–24^ To achieve electrospinnability with lignin, a binder polymer has often been added to the solution. This includes polyacrylonitrile (PAN),^25–27^ poly­(ethylene oxide) (PEO),^13,15,22^ poly­(vinyl alcohol) (PVA),^12,16–18^ polyvinylpyrrolidone (PVP),^11,28^ cellulose acetate (CA),^19,29^ polycaprolactone (PCL),^30,31^ poly­(ethylene terephthalate) (PET),^14^ polylactic acid (PLA),^23^ and poly­(acrylonitrile-*co*-methyl acrylate) (PAN–MA).^32^ Many of these polymers are currently derived from petroleum-based processes. In addition, toxic solvents are often used, such as *N,N-*dimethylformamide (DMF).^33–37^ Furthermore, the binder polymer often appears in relatively high concentrations in the electrospun fibers. The binder polymer was avoided by using coaxial electrospinning with an external flow of glycerol.^38,39^ However, the added complexity of this approach should be carefully assessed before its industrial scale-up. Due to these reasons, further studies are needed to minimize the use of a binder polymer in single-needle electrospinning processes.

Organosolv lignin-based electrospun fibers have been useful for applications where their sulfur-free content is an advantage; for example, in membranes for water treatment.^19^ Carbonized organosolv fibers have also been applied as a highly porous carbon support for catalysts, improving dispersion and catalyst activity.^20^ Energy storage applications, such as supercapacitors^21,22^ and lithium-ion batteries,^23^ have also greatly benefited from the high carbon content of organosolv lignin, where microporosity in the fibers contributes to faster ion transfer and improved electrochemical performance.^24^

The need for specialty organosolv nanofibers in these and other applications calls for systematic studies focused on the electrospinning process. Specifically, more work is needed (i) to identify compositions and robust process conditions to achieve uniformly sized nanofibers and (ii) to understand the underlying fiber formation mechanism. Although the internal structure of the as-spun fibers is key to understand the formation mechanism, it has appeared only rarely and for already carbonized fibers.^40^ Neither have previous studies addressed the stability of the jet emission as a requirement for fiber size uniformity. Finally, no fiber formation mechanisms have been discussed.

In this work, we conducted a study of single-needle electrospinning with two different sources of organosolv lignin (OL) (hardwood and softwood) with poly­(ethylene oxide) (PEO) as the binder polymer in a water-based solvent. Organosolv lignin has been electrospun in combination with PEO as the binder polymer in water.^24^ PEO is biocompatible and has been widely used in electrospinning studies for decades. However, since PEO is of fossil-fuel origin, here we aimed to identify solution compositions that lead to stable electrospinning while maximizing the lignin/PEO ratio. Different molecular weights of PEO were also considered to further reduce the needed PEO amount. We address the influence of solution composition on the morphology of the obtained fibers, examining both their outer and inner structures. The internal morphology of the fibers was characterized by the FIB-FESEM technique. To certify the stability of the electrospinning, the jet formation process was continuously imaged using high-speed video. Finally, a fiber formation model is proposed.

## Materials and Methods

### Materials

Poly­(ethylene oxide) (PEO) with viscosity-averaged molecular weights *M*_ν_ of 600,000 g mol^–1^ (PEO-600), 1,000,000 g mol^–1^ (PEO-1000), and 5,000,000 g mol^–1^ (PEO-5000) (CAS number: 25322-68-3) were purchased from Sigma-Aldrich. Softwood organosolv lignin (SOL, spruce wood, *M*_*n*_ 1,030–1,740 g mol^–1^ and *M*_*w*_ 4,090–10,490 g mol^–1^) and hardwood organosolv lignin (HOL, eucalyptus wood, *M*_*n*_ 1,030–1,060 g mol^–1^ and *M*_*w*_ 2,960–3,400 g mol^–1^) were kindly provided by the Fraunhofer Center for Chemical-Biotechnological Processes (CBP), Germany. Sodium hydroxide pellets (NaOH, CAS number: 1310-73-2) were purchased from Fisher Scientific. Deionized (DI) water was also used. All chemicals were used as received, without further purification.

### Solution Preparation

Sodium hydroxide (NaOH) pellets were dissolved in deionized (DI) water to prepare a 0.5 M NaOH aqueous solution. PEO was then dissolved in 0.5 M NaOH under vigorous stirring (300–500 rpm) at room temperature for 24 h. Subsequently, organosolv lignin was added to the PEO/0.5 M NaOH solution and dissolved under the same conditions for 24 h. The solutions were characterized rheologically and used within 1 week of preparation to produce electrospun fibers. Lignin/PEO composition solutions are presented in Figure S1.

### Fiber Preparation

Full setup and equipment details can be found in the Supporting Information file (under *Electrospinning setup and equipment details*). Briefly, the electrospinning process was carried out inside of a custom-made sealed chamber through which air flowed continuously, at near-room pressure. The solution was pumped at a constant flow rate *Q* (0.5 mL/h) from a plastic syringe to the electrospinning needle. A high DC voltage *V* (10–20 kV) was applied to the 22-G needle (ID = 410 μm, OD = 720 μm) from a high-voltage power supply. The fibers were collected on a solid cylinder placed at a distance *H* of 21 cm below the needle, which rotated at 1100–1200 rpm and was wrapped with aluminum foil. A ring-like back electrode set at *V* was used to reduce the rotation angle of the jet and gain efficient fiber collection. All electrospinnable compositions resulted in homogeneous coverage of the foil with fiber. Relative humidity (RH: 35–50%) and temperature (*T*: 21–25 °C) in the chamber were continuously monitored (Figure S1). The Taylor cone and jet emission region were imaged by high-speed video.

### Rheological Characterization of the Solutions

A Discovery Hybrid Rheometer (HR) 20 (TA Instruments, UK) was used with a cone and plate geometry of 40 mm diameter and 2° angle. Approximately 600 μL of solution were placed between the geometries (51 μm of truncation and 2.55 μm of trim gap). Steady-shear viscosity data were obtained via shear rate sweeps (*flow sweep*) between 1 and 1000 s^–1^. The steady-shear viscosity data were fitted using the Cross model, $\frac{\eta -\eta_{\infty }}{\eta_{0}-\eta_{\infty }}=\frac{1}{1+{\left(\lambda \mathrm{\gamma ̇}\right)}^{n}}$ , where η_0_ and *η*_∞_ are the zero- and infinite-shear viscosities, respectively, *n* is the power law index +1, and λ is a characteristic time-scale in the empirical Cross model, its inverse, λ^–1^, provides the shear rate at which the viscosity departs from its Newtonian plateau and shear-thinning becomes significant. Viscoelastic data were obtained from Small Amplitude Oscillatory Shear (SAOS) tests which were performed via small amplitude 1% strain, within the linear viscoelastic region, over the frequency range 1–20 Hz. The crossover relaxation time τ_*c*_ was calculated as τ_*c*_ = 1/ω_*c*_, where ω_*c*_ is the crossover angular frequency at which the storage $G^{\prime }\left(\omega \right)=A^{\prime }\omega^{n^{\prime }}$ and loss moduli $G^{\prime \prime }\left(\omega \right)=A^{\prime \prime }\omega^{n^{\prime \prime }}$ intersect, i.e., $G^{\prime }\left(\omega_{c}\right)=G^{\prime \prime }(\omega_{c})$ . All rheology tests were conducted at 25 °C with a soak time of 180 s, and typically (at least) three repetitions were performed for each precursor solution to obtain these parameters. Further details can be found in the SI file.

### Morphological and Structural Characterization of the Fibers

Two scanning electron microscopes (SEM) were used to image the electrospun fibers: a Quanta 600 and, for high-resolution imaging, a dual beam Scios 2 field emission scanning electron microscope (FESEM), both from FEI Company. Before imaging, the samples were gold coated for 90 s at 30 mA (∼14 nm estimated coating thickness) using a sputtering machine (Quorum Q150T S plus). The dual beam FESEM was equipped with a gallium-focused ion bean (FIB), which was used to mill the fibers to investigate their internal structure. Before FIB milling, the samples were Pt-coated in situ in two steps, first using electron beam-induced Pt deposition (dark gray layer), followed by ion beam-induced Pt deposition (light gray layer). For the sample milling, the ion beam energy and the probe current were 30 kV and 1 nA, respectively. Fiber diameters (sizes) were obtained from the SEM images, using ImageJ software (version 1.54g), and are reported for all electrospinnable conditions in Table S5.

X-ray diffraction (XRD) measurements were made using a Bruker-AXS D8-Advance diffractometer with vertical theta–theta goniometer, incident- and diffracted-beam Soller slits of 2.5°, a fixed 0.5° receiving slit and an automatic air-scattering knife on the sample surface. The angular 2θ range was between 5 and 80°. Data were collected with an angular step of 0.02° at a step/time of 0.5 s. Cu Kα radiation was obtained from a copper X-ray tube operated at 40 kV and 40 mA. Diffracted X-rays were detected with a Pσ detector LynxEye-XE-T with an opening angle of 2.94°. Sample was deposited on a low-background support (Si (510)). The diffractograms were interpreted using software DIFFRAC.EVA 6.0 from BRUKER.AXS and the PDF-2 database (2022 release) from ICDD (International Center for Diffraction Data).

All measured parameters are reported as the average ± standard deviation.

## Results and Discussion

### Solution Compositions Leading to Stable Electrospinning

The prerequisite for electrospinning stability is the continuous and regular emission of a single liquid jet or ligament from the electrospinning needle. High-speed video showed a Taylor cone meniscus undergoing a perfectly stable rotational motion around the needle axis (Figure 1). This rotational motion was found in all but one composition (Figure 1d). The rotational motion of the jet is more fully described in the Supporting Information file (Figure S2). Both situations (rotation and nonrotation) lasted the entire collection, typically several hours. Jet whipping happened further downstream in all cases, and allowed the fiber to disperse over the entire width of the cylindrical collector.

**Figure 1:**
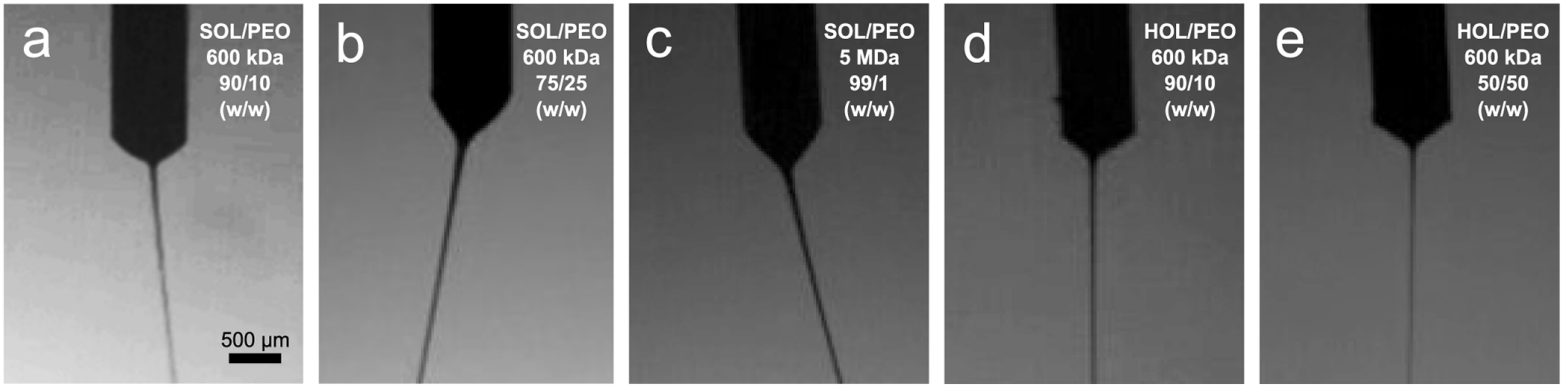
High-speed video frames of example cone-jets formed at the end of the electrospinning needle (cylindrical shadow, OD = 720 μm), for various solution compositions: 12 wt % 90/10 SOL/PEO-600 (a), 10 wt % 75/25 SOL/PEO-600 (b), 14.5 wt % 99/1 SOL/PEO-5000 (c), 12 wt % 90/10 HOL/PEO-600 (d), 8 wt % 50/50 HOL/PEO-600 (e), all in 0.5 M NaOH. The jet rotated in (a–c,e) but not in (d). Scale bar applies to all panels.

The composition parameters studied include the lignin/binder polymer ratio, the total solution concentration, the source of lignin (softwood and hardwood, with slightly distinct molecular weights) and the molecular weight of the binder polymer (PEO). The reason for a binder polymer is as follows. The entangling of long linear polymeric chains is believed to be a prerequisite for electrospinning by causing viscoelasticity in the solution. This can be achieved easily by dissolving linear polymeric chains at a concentration that is high enough to give enough entanglements.^41,42^ The condition for spinnability has been expressed as requiring an average number of entanglements per chain *n*_*e*_, known as *entanglement number* exceeding about 3.5 for the case of a polymer in a good solvent with nonspecific polymer–polymer interactions.^43,44^ A spinnability criterion based on *n*_*e*_ is convenient since *n*_*e*_ can be computed easily as $n_{e}=\frac{\phi_{p}M_{w}}{M_{e}}$ , where ϕ_*p*_ is the polymer volume fraction, *M*_*w*_ its weight-average molecular weight, and *M*_*e*_ the average molecular weight between entanglement junctions in the polymer melt. Since ϕ_*p*_ is less than unity, *M*_*w*_ must exceed *M*_*e*_. The typically low molecular weight of lignin after extraction (3–10 kDa) rules out the formation of entanglements among the lignin molecules. Therefore, a binder polymer with *M*_*w*_ ≫ *M*_*e*_ is commonly included to electrospin lignin.^45,46^ In our case, PEO’s with *M*_ν_ of 600 kDa (PEO-600) and higher are used. In the absence of lignin, a minimum of 1.3 wt % of PEO-600 would theoretically produce enough polymer chain entanglements to allow the production of fibers (see SI file).

The main tested compositions are represented in the ternary plot of Figure 2 and Table S1 (refer to the SI file for an interpretation aid). The different symbol shapes indicate whether each composition could be electrospun stably (black diamonds) or not (red crosses). Each point corresponds to two compositions, for SOL and HOL. In addition, several regions are identified according to whether the collected morphologies were *Fibers*, *Beaded fibers*, *Beads with incipient fibers*, or *Beads*. The transitions between the regions on the solvent axis (zero OL) are based on the critical *n*_*e*_ values estimated for PEO-600/water (see the SI file).

**Figure 2:**
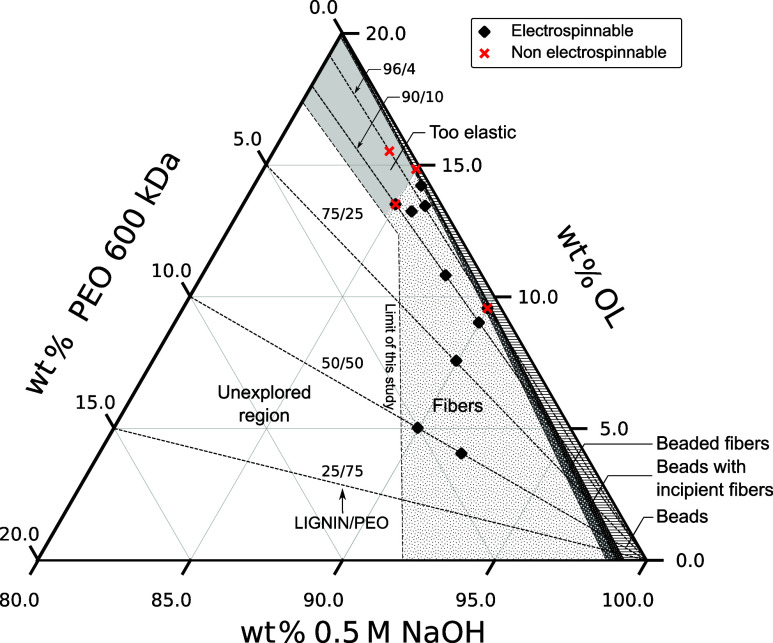
Ternary plot of precursor solution compositions showing electrospinnability domains considering the weight %’s of binder polymer (PEO 600 kDa), organosolv lignin (OL) and solvent (0.5 M NaOH in water). Each point represents two compositions depending on OL source (SOL, HOL). Diamond symbols represent stable electrospinnable compositions, while red crosses indicate nonelectrospinnable compositions. Regions corresponding to different solid morphologies are indicated as *Fibers*, *Beaded fibers*, *Beads with incipient fibers* and *Beads*. The intersections between these regions and the solvent axis (zero lignin content) have been computed following Shenoy et al.^43^ (see SI file). The *Too elastic* region, unsuitable for electrospinning, is identified experimentally. The *limit of study* line defines the scope of our exploration.

Our stable-electrospinning compositions covered a roughly vertical band of compositions, defined by increasing values of both the lignin-PEO ratio and the total polymer concentration (for OL plus PEO). The interdependence between these two parameters in obtaining stable electrospinning arises from the need to maintain enough viscoelasticity to compensate for the loss of PEO chain entanglements as the concentration of PEO is reduced. In other words, we could minimize the concentration of PEO (thus PEO chain entanglements) by increasing the concentration of lignin in the solution. Meanwhile, at high enough polymer concentrations, we encountered a limit in which a thick filament traveled straight to the collector, possibly due to excessive elasticity. We denote this region in the ternary plot as the *Too elastic* region. As the polymer concentration is further increased in this region, it became impossible to even form a Taylor cone.

Within the *Fibers* region of the ternary plot, the compositions were chosen mostly along three paths. In one, the OL/PEO weight ratio is varied at a constant total polymer concentration of 10 wt %. In another path, the total polymer concentration is varied at a constant OL/PEO weight ratio of 90/10. Finally, the polymer concentration and lignin/PEO ratio are varied simultaneously along a roughly “vertical” path, spanning wider parameter ranges (∼8 to 14.5 wt % and 50/50 to 98/2, respectively). Figure 2 shows that for the stable compositions (diamond symbols) the OL/PEO ratio could be increased beyond 90/10 if the total polymer concentration was increased beyond 10 wt %. The ratio 98/2 is the absolute highest at which stable electrospinning became possible with PEO 600 kDa, producing *beaded fibers*.

Table 1 summarizes the main rheology data for OL/PEO-600 solutions. The complete data set can be found in Table S3. SAOS tests were performed within the linear viscoelastic regime under shear flow, which differs from elongational flow found in electrospinning, but still provides essential information on solution structure and solute interactions. The main trends are as follows. The viscous parameters, zero-shear viscosity η_0_ and the characteristic shear-thinning time λ, increase (nonlinearly) with increasing total polymer concentration or decreasing lignin/PEO ratio. Note that the values of both η_0_ and λ are systematically lower for HOL than for SOL. This suggests a weaker PEO–HOL interaction, possibly from the lower molecular weight than SOL.

**Table 1:** Rheology Parameters of OL/PEO-600 Solutions in 0.5 M NaOH at 25 °C: Zero-Shear Viscosity (*η*_0_), Characteristic Time for the Onset of Shear-Thinning (*λ*), and Characteristic Crossover Relaxation Time (*τ*_*c*_)­^b^

Lignin	OL/PEO wt ratio	Poly. (wt %)	OL (wt %)	PEO (wt %)	η_0_ (mPa s)	λ (ms)	τ_*c*_ (ms)
SOL	50/50	8.1	4.1	4.0	1,874 ± 54	14.6 ± 0.5	3.2 ± 0.3
		10.0	5.0	5.0	5,768 ± 407	34 ± 2	5.1 ± 0.2
	75/25	10.1	7.5	2.6	834 ± 39	8.9 ± 0.3	2.3 ± 0.6
	90/10	10.0	9.0	1.01	82 ± 4	1.4 ± 0.4	–
		12.1	10.8	1.23	299 ± 6	6.02 ± 0.04	1.6 ± 0.3
		15.0	13.5	1.5	2,522 ± 304	34 ± 3	4.0 ± 0.6
	92/8	14.4	13.2	1.19	447 ± 23	7.8 ± 0.3	2.4 ± 0.4
	96/4	10.0	9.6	0.46	17.8 ± 0.5^a^	–	–
		14.0	13.4	0.57	147 ± 36	3.5 ± 0.8	2.6 ± 1.9
	98/2	14.5	14.2	0.30	26.7 ± 0.2	7.43 ± 0.04	–
	100/0	7.5	7.5	0.00	1.44 ± 0.07^a^	–	–
HOL	50/50	8.1	4.1	4.0	862 ± 29	6.3 ± 0.2	2.3 ± 0.1
		10.0	5.0	5.0	2,210 ± 290	11 ± 2	2.7 ± 0.4
	75/25	10.0	7.5	2.5	218 ± 20	2.3 ± 0.3	2.1 ± 0.6
	90/10	10.0	9.0	1.04	34 ± 5^a^	–	–
		12.0	10.8	1.22	66 ± 1	1.3 ± 0.1	–
		15.0	13.5	1.51	375 ± 3	3.4 ± 0.5	1.9 ± 0.3
	92/8	14.3	13.2	1.15	105.3 ± 0.4	2.3 ± 0.1	0.88 ± 0.09
	96/4	10.2	9.6	0.44	7.4 ± 0.0^a^	–	–
		14.0	13.4	0.58	56 ± 10^a^	–	–
	98/2	14.5	14.2	0.30	8.6 ± 0.5^a^	–	–
	100/0	7.5	7.5	0.00	1.36 ± 0.04^a^	–	–

a Solution has Newtonian behavior.

b Used in the least-squares fitting (see text).

For most compositions, the relaxation time estimated from τ_*c*_ is within the millisecond range, which is comparable to the characteristic initial jet deformation times reported in electrospinning literature (∼10^–3^–10^–2^ s).^47^

An interesting question is why spinability is possible for the fibers with high lignin content. In Figure 2, this is the region defined by OL/PEO ≥ 90/10 and polymer concentration between 10 and 14.5 wt % where it was possible to electrospin under conditions of lower than theoretical PEO chain entanglement numbers, producing fibers or beaded fibers. We have thus analyzed the solute concentration dependence of the specific viscosity η_*sp*_ = (η_0_ – η_*s*_)/η_*s*_, where the solvent viscosity η_*s*_ for 0.5 M NaOH is 0.997 mPa s (*T* = 25 °C).^48^ In this region, the specific viscosity data can be fitted to η_*sp*_ = A­[*Polymer*]^*B*^, where [*Polymer*] stands for the total polymer concentration, and *A* and *B* are linear functions *A*_1_ + *A*_2_ *x* and *B*_1_ + *B*_2_ *x* of the polymer ratio *x* = [PEO]/[OL]. The exponent *B* ranges between 4.2 and 6.1 for SOL and between 6.1 and 7.5 for HOL over the range of 98/2 ≤ *x* ≤ 90/10 (see SI file for more details). This is consistent with a entangled network, consistent with electrospinnability.^41^ Since this occurs in the region of highest lignin and lowest PEO concentrations, this result suggests that the small lignin molecules promote entanglements or connections between the PEO chains, thereby producing a sufficiently developed network.

### Fiber Morphology Versus Composition

Figure 3 shows the dependence of fiber morphology on the OL/PEO ratio with a constant total polymer concentration of 10 wt %. Beaded fibers were encountered at ratios of 96/4 for SOL (Figure 3(A4)) and 90/10 for HOL (Figure 3(B3)). At lower OL/PEO ratios, we obtained fibers. The fibers are submicrometric and are systematically thinner for HOL than SOL, correlating with the lower viscosity of the HOL solutions (Table 1). For the highly viscous 50/50 cases, the flow rate had to be reduced to reach stable conditions, and this may help explain why the SOL fibers were thinner at that ratio than at 75/25 (Table S1). Conversely, the 96/4 HOL/PEO solution had very low viscosity and Newtonian character. It therefore led to an unstable condition in which the Taylor cone oscillated axially (red cross in Figure 2). The frequency of the oscillation could be reduced by lowering the flow rate, but the instability persisted even at 10 times lower rate (0.05 mL/h). At this condition, the collected morphology was beads with incipient fibers with polydisperse sizes (Figure 3(B4)), consistent with the so-called spindle mode in electrospraying.^49^

**Figure 3:**
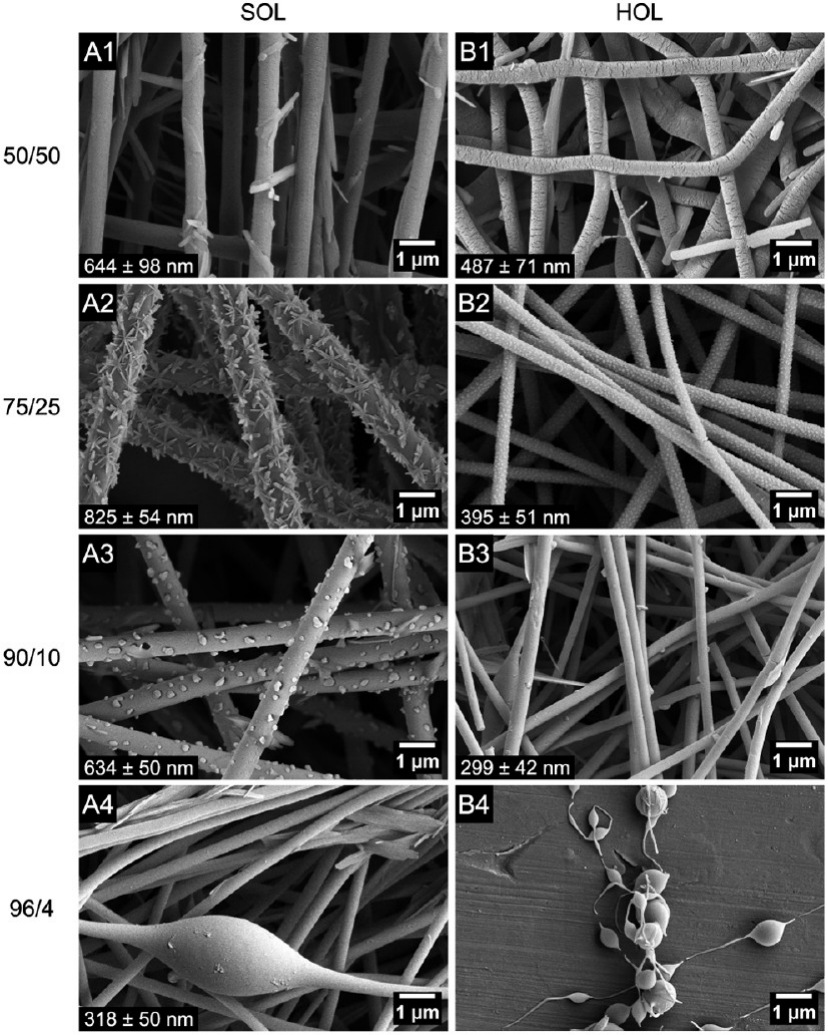
FESEM top views of SOL (A panels) and HOL (B panels) fibers electrospun from feed solutions with total polymer concentrations of 10 wt % and different lignin/PEO-600 weight ratios (1) 50/50, (2) 75/25, (3) 90/10, (4) 96/4. *Q* = 0.5 mL/h, except for (A1,B1) 0.1 mL/h, (A4) 0.2 mL/h, and (B4) 0.05 mL/h.

The total polymer concentration was then raised beyond 10 wt % while keeping a constant lignin/PEO ratio of 90/10 (Figure 2). For SOL, the solution became non electrospinnable at 15 wt % polymer concentration, leading to a nonwhipping jet with a straight path to the collector which deposited a wet mass (Figure S5). At this concentration η_0_ had increased rapidly (Table 1), suggesting that the viscosity was excessive. The HOL solution at that concentration was still stable, as is much less viscous (by a factor of ∼7). Figure 4 shows the dependence of the HOL fibers with polymer concentration. The fibers average diameters increase with polymer concentration, correlating with increasing η_0_. The cross sections obtained by FIB-FESEM are shown in the insets of Figures 4 and S9. They demonstrate that the fibers were circular and contained numerous inner voids. The inner structure evolves from apparently hollow to nearly compact with occasional voids as concentration increases, whereas at the intermediate concentrations the fibers contained bubbles.

**Figure 4:**
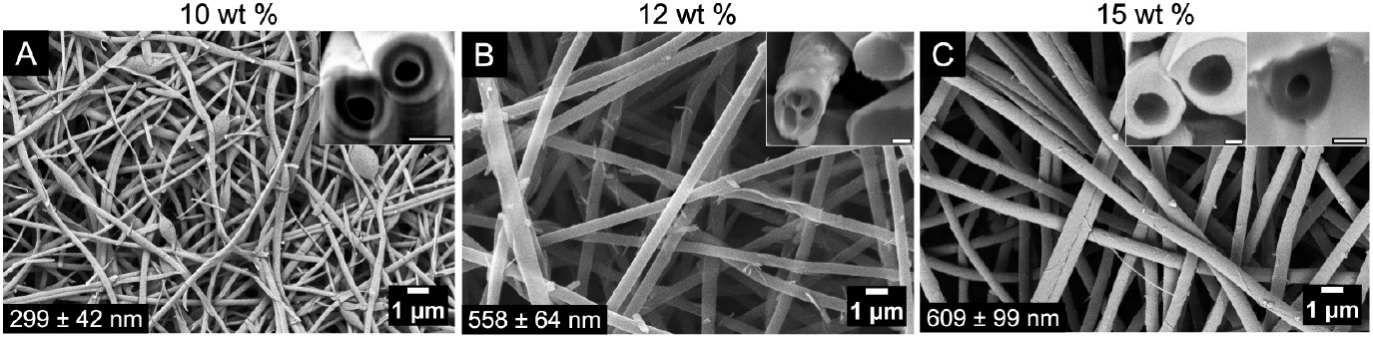
FESEM top views and FIB-FESEM cross sections (insets) of HOL fibers electrospun from feed solutions with 90/10 lignin/PEO-600 weight ratios and total polymer concentrations of: (A) 10 wt %, (B) 12 wt %, (C) 15 wt %. Inset scale bars = 200 nm. Additional FIB-FESEM images in Figure S9.

The experiments of Figures 3 and 4 helped us to identify upper limits in the composition parameters separately. To maximize the lignin/PEO ratio in the fiber, higher values of these parameters were explored in combination. Figure 5 displays FESEM images of (A) SOL and (B) HOL nanofibers with PEO-600, for lignin/PEO weight ratios ranging from 50/50 to 96/4, while the total polymer concentration is increased. Cross sections of FIB-milled fibers are shown in the inset for each condition. Again, we find circular fibers that contain numerous inner voids and particles or structures attached to the outside of the fibers, especially for the case of SOL. We also see that the fiber diameters are under 1 μm (Table S5). The average fiber diameters do not show a monotonous variation with composition but, as before, are generally smaller for HOL than for SOL compositions.

**Figure 5:**
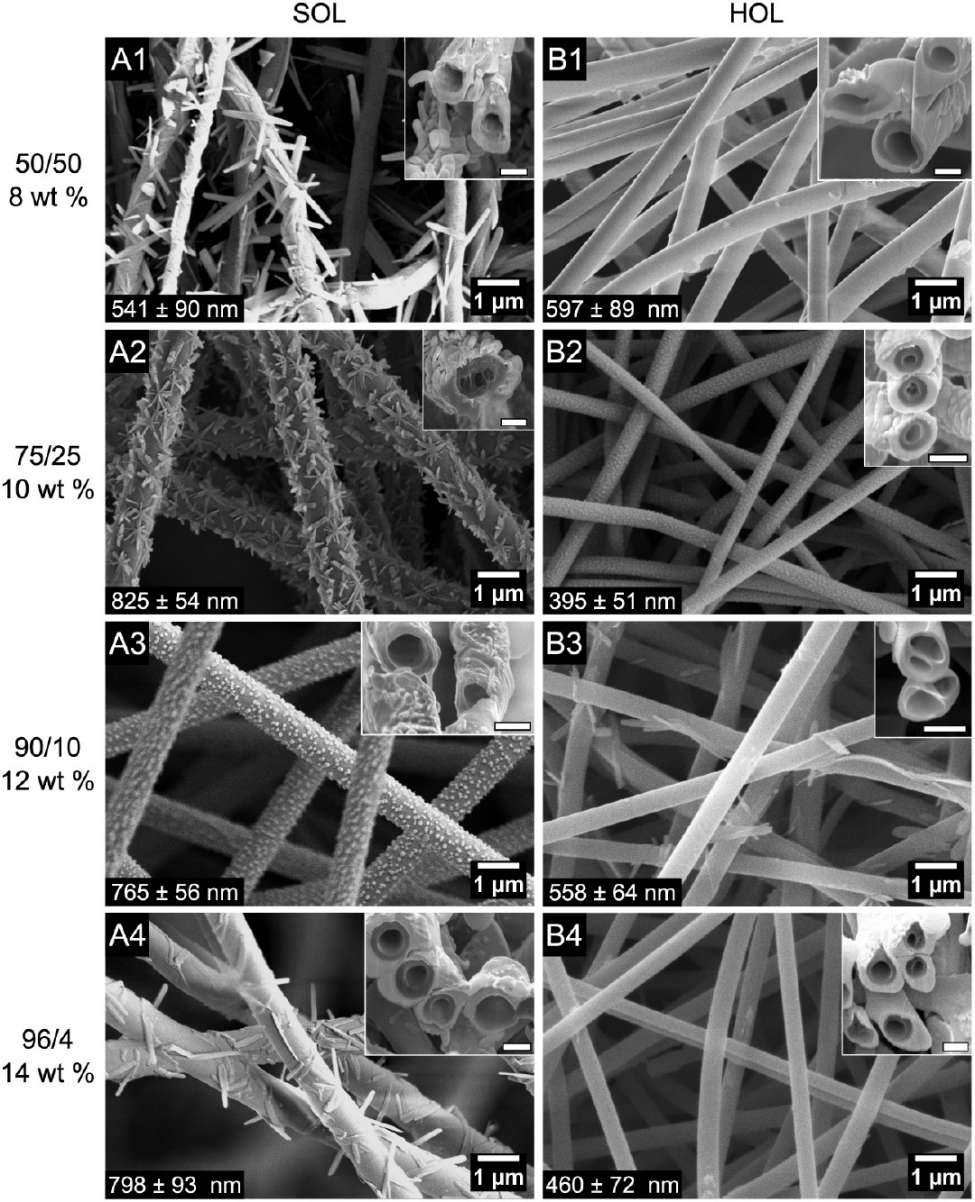
FESEM top views and FIB-FESEM cross sections (insets) of SOL (A panels) and HOL (B panels) fibers electrospun from feed solutions with different lignin/PEO-600 weight ratios and total polymer concentrations of: (1) 50/50, 8 wt %; (2) 75/25, 10 wt %; (3) 90/10, 12 wt %; (4) 96/4, 14 wt %. Inset scale bars = 500 nm. Fiber diameter is presented at the left bottom. (More information in Table S5. Additional FIB-FESEM images in Figure S10.).

Regarding the internal structure of the fibers in Figure 5 (also Figure S10), all samples had voids. For the composition with the lowest lignin content (50/50 lignin/binder), the HOL fibers appear hollow while the SOL fibers have either one large void or a porous core (small voids, Figure S10­(A1)­(B1)). SOL at 75/25 ratio also has a porous core. For the other compositions (with dominant lignin mass fraction), the sections show either voids of different sizes or compact sections (without voids) within the same sample. To further investigate this, we sectioned fibers at longitudinal inclinations for the 75/25 and 90/10 HOL/PEO-600 cases (Figure 5(B2)­(B3)). The result is shown in Figure 6, where the voids are shown to be bubbles or several partially merged bubbles. Finally, we note that the heterogeneous nature of the voids contrasts with the smooth cylindrical shape of the fibers (Figure 5). This strongly suggests that during the fiber formation the outer shape of the fiber is established before the inner voids.

**Figure 6:**
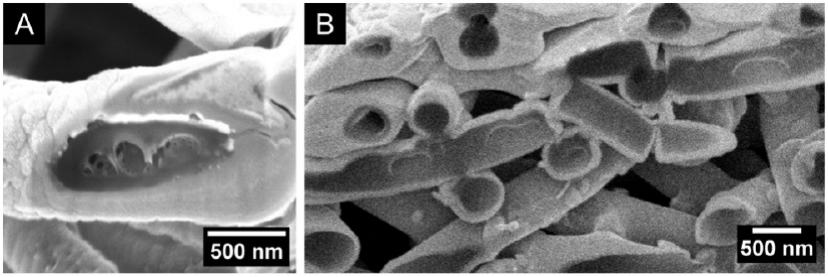
HOL/PEO-600 fibers imaged by FESEM after FIB-milling, electrospun from feed solutions with different lignin/binder polymer ratios and concentrations of (A) 75/25, 10 wt %; and (B) 90/10, 12 wt %.

The above experiments show that SOL and HOL fibers with a highest OL/PEO-600 ratio of 96/4 could be stably produced from a solution at 14 wt % polymer concentration (Figure 5). However, at the very proximal composition of 14.5 wt % and 98/2 lignin/PEO ratio, beaded fibers were found for both SOL and HOL (Figure S4). Therefore, 14 wt % represents the maximum processable concentration for a molecular weight of 600 kDa.

To further reduce the content of binder polymer (PEO), its molecular weight was increased to *M*_ν_ 1 MDa. This permitted the stable production of fibers at OL/PEO 98/2 and 14.5 wt % total polymer concentration. Figure 7 shows the fibers, which were significantly thicker for SOL than HOL, with occasional voids in the former and abundant small bubbles in the latter. This difference may be attributed to the limit of entanglement of each solution, with the HOL solution being closer to the threshold of *complete fiber formation*.

**Figure 7:**
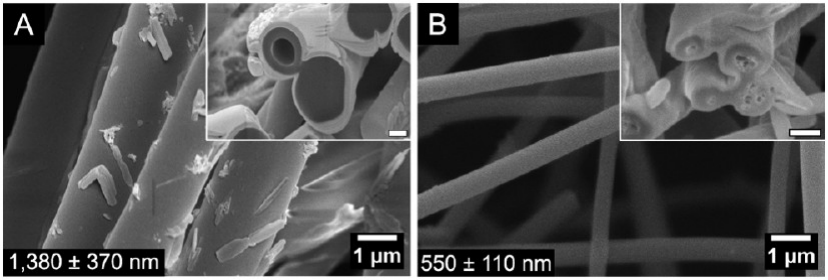
FESEM top views and FIB-FESEM cross sections (insets) of SOL (A) and HOL (B) fibers electrospun from lignin/PEO-1000 98/2 weight ratios and total polymer concentration of 14.5 wt %. Inset scale bars: (A) 500 nm and (B) 200 nm. Fiber diameter is presented at the left bottom.

The external structures on the surface of the fibers in Figures 3 and 5 vary in shape depending on the composition. They are more visible for the SOL/PEO fibers, but are also present on the HOL/PEO ones. The acicular structures on SOL fibers (Figure 3(A1) and (A2), and Figure 5(A1), (A2), and (A4)) suggest a crystalline precipitate from the dissolved NaOH. At the intermediate SOL/PEO ratio of 90/10, the external structures were granular (Figure 3(A3) and Figure 5(A3)), which might be amorphous or very small crystals. To confirm that the variability in morphology is not due to chance errors in experimentation, numerous independent experiments were carried out for the SOL/PEO weight ratio of 90/10 (Figures S7 and S8). In addition, the same granular structure can be found in the literature^50^ for the same composition although for a different softwood source and molecular weight distribution. This suggests that the granular morphology is insensitive to those parameters, but rather seems to be connected to the lignin/PEO ratio.

Finally, we compare our fiber size (Table S5) with previous studies on organosolv lignin electrospun solutions. Some researchers used coaxial electrospinning to obtain fibers without binder polymer below 1 μm, despite significant size polydispersity.^38,39^ Other studies obtained fibers in the range of 1–1.5 μm using PEO with a high lignin/binder ratio of 95/5–99/1, but used DMF as a solvent.^36,37^ Similarly, other works yielded fibers of ∼1 μm using PAN and DMF with a ratio of 70/30.^33,34^ In sum, our work reports the thinnest size-monodisperse organosolv fibers which combine high lignin/binder weight ratios (96/4) and fiber widths well below 1 μm.

### EDX and XRD Analyses of the External Morphologies

EDX and XRD analyses provided evidence of the different chemical natures between the nanocrystal and the neat fiber surface, thereby supporting the idea of Na_2_CO_3_ formation. Figure 8(A) shows the EDX analysis of electrospun fibers from SOL/PEO 75/25 10 wt % total polymer solution in 0.5 M NaOH. EDX spectra are obtained at two positions on the fiber corresponding to a nanocrystal and neat fiber surface (away from the nanocrystal).

**Figure 8:**
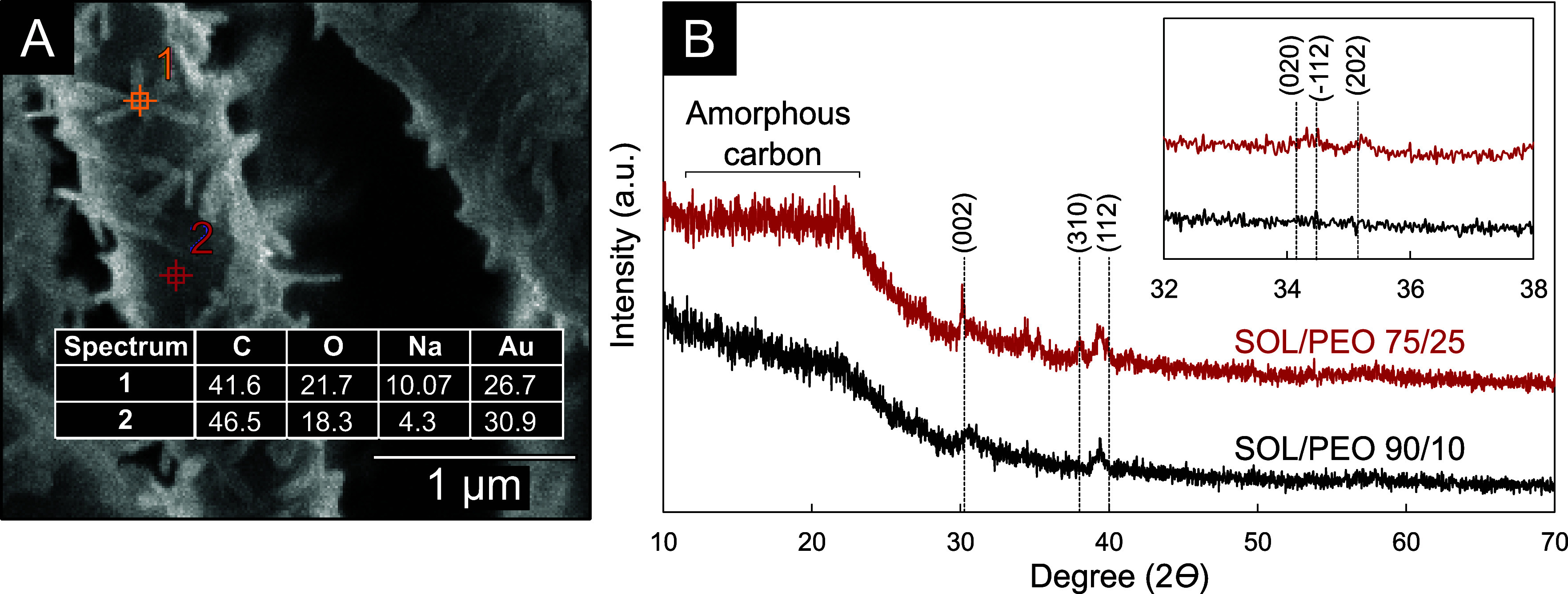
(A) EDX for two positions on an electrospun fiber from the solution of SOL/PEO-600 75/25 at 10 wt % total polymer in 0.5 M NaOH. The atomic percentages (AP) are presented in the inset. The AP for gold is attributed to the sputtered coating of this material. (B) XRD spectra of electrospun fibers from a 12 wt % total polymer SOL/PEO-600 90/10 and a 10 wt % total polymer SOL/PEO-600 75/25 in 0.5 M NaOH. The Miller indices are for the main crystalline planes for Na_2_CO_3_.

The atomic percentages (AP) obtained from the spectra are presented in Figure 8(A). The Na content was significantly higher for the beam aimed at the nanocrystal (Spectrum 1) than at a crystal-free zone (Spectrum 2). Although the AP’s for this element (or any other) are not perfectly localized values (as the electron beam penetrates significantly into the sample), we can certify that the nanocrystalline structures present on the surface fiber contain significantly more Na than the rest of the fiber. The O/Na ratio in Spectrum 2 (crystal-free zone) is 4.3, which is much higher than expected from NaOH or Na_2_CO_3_. On the other hand, the O/Na ratio in Spectrum 1 (nanocrystal) is 2.1. This value supports the existence of Na_2_CO_3_ or NaOH, for which the O/Na ratio is 1.5 and 1.0. These values are expected to be lower than 2.1 because of the penetration of the electron beam.

To investigate which of the two crystalline compounds is present, XRD analyses were performed on the fibers from the conditions SOL/PEO 90/10 and SOL/PEO 75/25 (Table S5). The XRD spectra are shown in Figure 8(B). Both samples exhibited a broad amorphous carbon hump around 20°, which can be explained by the amorphous nature of the lignin fibers. The signal-to-noise ratio of the spectra was quite low, due to the small absolute amount of Na_2_CO_3_ in the samples. The signal was overall very weak, and only some of the characteristic peaks for Na_2_CO_3_ corresponding to the (002), (020), (−112), (310), (112), and (202) planes were expected at 30.24°, 34.16°, 34.47°, 35.15°, 38.04° and 39.98°, respectively (PDF-77-2082). Peaks at some of those angles are visible for the case of SOL/PEO 75/25. This can be explained by the larger fraction of crystallized matter in this case (Figure 5(A2) and (A3)). Furthermore, the peak at 39.4° (between the (310) and (112) peaks) is equally intense in both spectra, and can thus be attributed to the particle holder used during the analysis, in this case the fibers. However, additional studies are required to confirm this hypothesis.

In sum, the external structures of the fibers are very likely made of Na_2_CO_3_.

### Mechanism of Fiber Formation

Based on the video and morphological evidence collected, four distinct stages of fiber formation can be identified, as shown in Figure 9. In stage 1, the strong electrical pull on the lignin/PEO solution creates the Taylor cone (TC) and the jet. Taylor cones do not always form in electrospinning, where often the jet develops more gradually. Here, a TC forms with a well-defined cone whose angle is close to the theoretical Taylor value (98.6°). This behavior is typical of Newtonian fluids,^51^ suggesting that solution elasticity is not a dominant factor in the conical region, where surface tension stresses are balanced by normal electrical stresses.^49^ From the tip of the cone a jet is emitted under the action of electrical stresses, experiencing a significant axial strain rate $\mathrm{\epsilon ̇}=\partial v_{z}/\partial z$ , $\mathrm{\epsilon ̇}=-2v_{z}\left(\partial d/\partial z\right)/d∼Q/d_{jet}^{3}$ of the order of 10^2^–10^3^ s^–1^ (where *d* is the width of a flow section, coinciding with *d*_*jet*_ in the jet zone). Such *ϵ̇* values are consistent with the electrospinning literature.^52–54^ The fluid responds to the high strain rate through *strain hardening* by developing a longitudinal stress σ = η_*e*_ *ϵ̇* where the extensional viscosity η_*e*_ (not determined here) rises transiently.^55^ This stress decays further downstream through viscoelastic relaxation.^47,56^ Water evaporation is negligible during this stage (see SI file).

**Figure 9:**
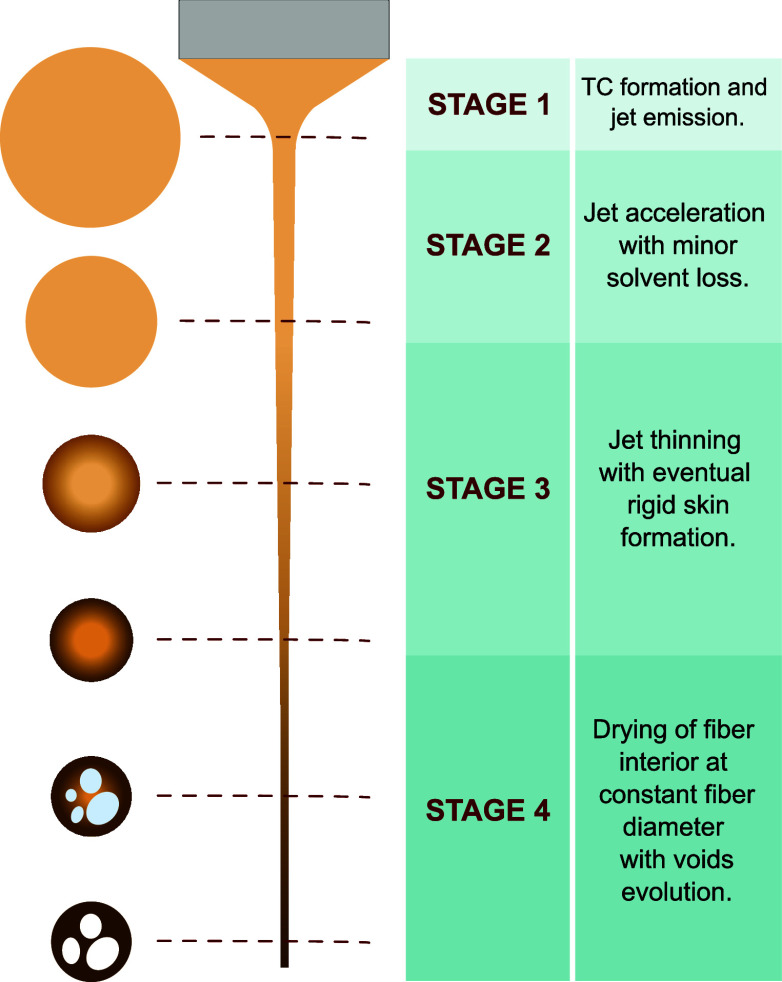
Proposed fiber formation stages. The brown tone represents the lignin concentration. For simplicity, the jet is shown to be straight and centered, although, in reality, it rotates with the Taylor cone. Also, the real jet is much longer and thinner and, further downstream, undergoes bending and whipping (somewhere in stages 2 or 3, not determined here).

When the jet is ejected, its diameter greatly exceeds that of the final fiber by 2 orders of magnitude (Figure 1). Therefore, the jet stretches significantly before it becomes a rigid fiber. Stage 2 comprises the initial electrostatically driven stretching of the ejected jet, without significant solvent evaporation. Initially, the strain (or elongational) rate of the jet is highest.^52,57^ Eventually, the longitudinal strain rate and the viscoelastic stresses decay through viscous relaxation.^47^ Also, as the jet thins, the air–liquid interface per unit volume increases, thus enhancing solvent evaporation. The solvent evaporation rate from a given length of jet *l* can be estimated as ${ṁ}_{vj}≃2\pi lDc_{v}^{\mathrm{sat}}\left(1-\mathrm{RH}\right)$ , where *D* and $c_{v}^{\mathrm{sat}}$ are the diffusion coefficient and saturation concentration of water vapor in air. The importance of the water evaporation from the jet is thus governed by the dimensionless ratio *ṁ*_*vj*_/(ρ*Q*), which for *l* = 1 mm equals 0.017. Thus, in the first cm of jet (for example) only about 17% of the initial flow has left as water vapor from the jet. As a result, radial concentration gradients of all species develop within the jet.

Therefore, a stage 3 can be identified with the development of significant solute enrichment at the jet surface, a decrease in the water evaporation rate (per unit solute mass) and, toward the end of the stage, the formation of a mechanically strong shell that surrounds a liquid interior. This picture agrees with the circularity and diameter uniformity of the fibers. The circular cross sections demonstrate that, during the drying process, a shell forms which supports the emerging hoop stresses without buckling.^58^ Similarly, the local uniformity in the fibers’ diameter (Figures 3–7) shows that the growth of voids within the fiber does not mechanically distort the shell. This suggests a picture in which the elastic modulus at the jet surface grows as the liquid evolves from a viscous state to, eventually, a glassy material. Meanwhile, the interior of the fiber still contains a significant amount of water (roughly as much as the volume of the voids). Similar mechanisms are believed to operate in the formation of spheroidal globular particles by electrospray.^44^

Hardening of the jet surface in stage 3 is also important for preventing beaded fibers, namely the growth of axisymmetric capillary waves along the liquid jet. The characteristic time for wave growth decreases with increasing viscosity and elasticity; but it is not infinite. Therefore, beads can be prevented only if the jet surface hardens fast enough. For example, beading is observed at 90/10 OL/PEO ratio and 10 wt % polymer concentration in Figure 3(A4,B3), for which the viscosities are relatively low (Table 1). However, beading disappears for compositions with greater viscosity, after either reducing the lignin/binder ratio (Figure 3) or increasing the total polymer concentration (Figure 4). In addition, normal electrical stresses may play a stabilizing role by reducing the growth rate of capillary waves, due to the high concentrations of Na^+^ and OH^–^ ions.^59–61^ This mechanism may explain why we get continuous fibers without beading from our solutions with relatively low viscosities at high lignin/PEO ratios, provided their total polymer concentration is high enough (Table S3).

Finally, stage 4 begins when the jet has become a fiber of constant diameter, comprising a substantially dry shell and a wet core. Water molecules diffuse from the fiber’s core to its surface, where they evaporate. They leave the core solution by diffusing either to and through the shell or to a nearby void, from where water molecules can cross the shell. These pathways were previously hypothesized also for solvent (water/ethanol) in PEO solutions in hollow fibers produced by coaxial electrospinning.^62^ Meanwhile, tiny voids nucleate at multiple sites, filling up with air that permeates through the vitreous shell. The voids forming in the fiber core move and aggregate, forming larger voids, until the fiber dries up completely. These motions are slow (due to viscosity), sometimes leading to partially merged voids (Figure 6). The interior structure depends on how fast the fiber dries up, which depends on the rheology. This is best seen in Figure 4, where the changes in the initial polymer concentration changes the viscosity by a factor greater than 10 (Table 1), while the HOL/PEO ratio is fixed, making the cases comparable. At the lowest polymer concentration (Figure 4(A)) implies faster jet thinning and drying, with stronger concentration gradients developing in the jet over a shorter time scale. This leads to earlier vitrification and to more water trapped within the fiber, and finally, a fiber that is apparently hollow. At the higher polymer concentrations (12 and 15 wt %; Figure 4(A,B)), the jet is more viscous. As it stretches more slowly, less interface per unit mass is generated, leading to slower evaporation and more solute diffusion within the fiber before vitrification takes place. This results in a thicker fiber and a more compact interior. Note that stage 4 could take place, partially or entirely, after the fiber reaches the collector. Nonetheless, the uniformity of the fibers’ width demonstrates that the fibers do not experience any deformations upon reaching the collector (such as breakup by the Rayleigh-Plateau/Weber instability^63^), and are therefore substantially dry and rigid when hitting the collector (Figure 9).

## Conclusion

We demonstrated the successful electrospinning of organosolv lignin-based nanofibers with very low binder polymer. Lignins derived from hardwood and softwood sources were utilized to produce fibers with internal voids and hollow structures, exhibiting diameters within the submicrometer range.

The rheological characterization of the solutions quantified the viscoelastic and non-Newtonian nature through characteristic relaxation time and shear-thinning behavior.

The stability parameters were investigated to study the jet rotational motion and determine the minimum amount of binder polymer required to produce fibers. It was possible to reduce the concentration of PEO in the fibers below the minimum theoretically required to achieve PEO chain entanglements in the solutions by increasing the total polymer concentration. For these solutions, the concentration dependence of the specific viscosity shows that the small lignin molecules promote entanglements between the PEO chains, thereby producing a sufficiently entangled network with high lignin content.

Operational parameters were studied to tune the morphological characteristics of the electrospun fibers, which exhibited narrow dispersion in the fiber diameters. In addition, internal voids and hollow structures were observed in all fibers. Some fiber surfaces revealed nanostructures with various morphologies identified as Na_2_CO_3_ by means of EDX and XRD.

A fiber formation mechanism was proposed on the basis of the observed internal structure of the as-spun fibers.

In conclusion, this work provides a comprehensive study on the feasibility of electrospinning as a promising technique for the fabrication of organosolv-lignin-based nanofibers with controlled morphological properties and internal porous structure. It highlights the complex interplay between the lignin and the binder polymer (here PEO), which is indispensable for achieving stable electrospinning and must be present in a small but finite concentration.

## Supplementary Material



## Notes

Additional experimental details, materials, and methods, including photographs and design of experimental setup (PDF)
