# Supplementary material for: Organosolv Lignin-Based Electrospun Nanofibers: Stable Compositions and Morphological Insights
Source: ACS Sustain Chem Eng. 2026 Jan 15;14(4):1873–83. doi: 10.1021/acssuschemeng.5c09295 (PMC12879301; doi:10.1021/acssuschemeng.5c09295)
Supplement: Supplementary file 1 [file sc5c09295_si_001.pdf]

Supporting Information

Organosolv Lignin-Based Electrospun Nanofibers: Stable Compositions and Morphological Insights

Paula Martínez Cánovas[a], Salvatore Cito[b], Francisco Medina[a], Joan Rosell-Llompart[a,c,*]

[a] *Department of Chemical Engineering, Universitat Rovira i Virgili, Tarragona, E-43007, Spain*

[b] *Department of Mechanical Engineering, Universitat Rovira i Virgili, Tarragona, E-43007, Spain*

[c] *Catalan Institution for Research and Advanced Studies – ICREA, Barcelona, E-08010, Spain*

[*] Corresponding author: E-mail address: joan.rosell@urv.cat

Number of Pages: 18

Number of Figures: 12

Number of Tables: 5

Tested solution compositions

The solution compositions that were tested for electrospinning stability are presented in Table S1. Compositional factors included organosolv lignin source (softwood (SOL), hardwood (HOL)), PEO molecular weight, total polymer concentration and lignin/PEO weight ratio. Instead of the last two, the table shows the concentration of the components (OL, PEO, and the solvent 0.5 M NaOH). This identification of the PEO-600 compositions in the ternary diagram. See the Methods section for more details.

Table S1. Tested solution compositions with outcome and needle voltage leading to stable process. (Needle voltage = V). Electrospinnable (E) or Non-electrospinnable (NE).

Lignin	PEO M_ν (kDa)	Lignin/PEO weight ratio	OL (wt %)	PEO (wt %)	0.5 M NaOH (wt %)	V (kV)	Stability (E/NE)	Morphology
SOL	600	50/50	4.1	4.1	91.9	15.0–17.0	E	Fibers
			5.0	5.0	90.0	15.0–16.0	E[a]	Fibers
		75/25	7.6	2.5	90.0	11.1–12.0	E	Fibers
		90/10	9.0	1.0	90.0	16.5	E	Fibers
			10.8	1.21	88.0	12.5	E	Fibers
			13.5	1.5	85.0	11.8	NE[b]	Accumulation
		92/8	13.2	1.11	85.6	15.0	E[c]	Fibers
		96/4	9.6	0.46	90.0	12.0	E[d]	Fibers with rare beads
			13.5	0.56	86.0	12.1	E	Fibers
			15.5	0.70	83.8	–	NE	Too elastic
		98/2	14.2	0.31	85.4	11.8	E	Beaded fibers
		99/1	14.9	0.16	85.0	–	NE[e]	Beads
	1,000	98/2	14.3	0.31	85.4	14.6	E	Fibers
	5,000	99/1	15.1	0.18	84.7	14.9–15.9	E	Fibers
		99.5/0.5	14.9	0.09	85.0	–	NE[e]	Beads
	–	100/0	22.7	–	77.3	–	NE[e]	Beads
HOL	600	50/50	4.0	4.0	92.0	18.0	E	Fibers
			5.0	5.0	89.0	20.0	E[a]	Fibers
		75/25	7.6	2.5	89.9	16.2	E	Fibers
		90/10	9.0	1.0	90.0	14.1	E	Fibers with rare beads
			10.9	1.20	87.9	12.0	E	Fibers
			13.5	1.5	85.0	13.3	E	Fibers
		92/8	13.2	1.15	85.6	9.1	E	Fibers
		96/4	9.6	0.44	89.9	17.1	NE[f]	Beads with incipient fibers
			13.5	0.57	85.9	14.0	E	Fibers
			15.5	0.65	83.8	–	NE	Too elastic
		98/2	14.3	0.30	85.4	15.9	E	Beaded fibers
		99/1	14.9	0.18	84.9	–	NE[e]	Beads
	1,000	98/2	14.2	0.31	85.5	14.6	E	Fibers
	5,000	99/1	14.7	0.18	85.1	11.4	E[g]	Fibers
		99.5/0.5	14.9	0.11	85.0	–	NE[e]	Beads
	–	100/0	22.8	–	77.2	–	NE[e]	Beads

[a] Q = 0.1 mL/h.

[b] Straight jet (without whipping) all the way to the collector, producing material accumulation.

[c] Q = 1.0 mL/h; studied at H = 20, 25 cm.

[d] Q = 0.2 mL/h.

[e] Explored V = 10 – 20 kV.

[f] Q = 0.05 mL/h; dripping mode.

[g] Fibers collected before completing solvent evaporation.

Electrospinning setup and equipment details

The electrospinning process was carried out with a custom-made set-up, consisting of a sealed chamber (cubic, 700 mm each side) plus a syringe pump and a high-voltage power supply (Figure S1(a)). Air (from the lab compressed air line) was admitted into the chamber and was exhausted into a laboratory fume-hood. Relative humidity (RH) and temperature (T) in the chamber were continuously monitored using a Vaisala HM34 probe inserted through the lateral wall of the chamber (Figure S1(a)). The solution was loaded into a plastic syringe (BD Plastipak™ luer-lock, 3 mL), and was then pumped at constant flow rate Q (0.5 mL/h) using a syringe infusion pump (New Era Instrument, N-300). The solution flowed out of the syringe into and through a capillary tube (Teflon™ tube 1/16 in OD, 300 μm ID) and then into the electrospinning needle, a flat-ended hypodermic needle (gauge 22; ID = 410 μm, OD = 720 μm) (Figure S1(b)). A high DC voltage V (10–20 kV) was applied to the needle and the back electrode from a high voltage power supply (Analog Technologies, Inc. AHVAC20KVR5MABT). A ring-like high voltage back electrode, also at V, was used to reduce the angle of jet rotation and gain efficient fiber collection. The fibers were collected on a solid cylinder (d = 7 cm) which was wrapped with aluminum foil and rotated at 1100 - 1200 RPM.The cylinder was placed a distance H of 21 cm below the needle. It was mounted on a microlathe (PROXXON DB 250), which had been customized by replacing its base with a heavy aluminum block to make room for the cylinder and provide mechanical stability, as shown in the Figure S1. This rotating collector had previously been used in our laboratory.[1] The Taylor cone and jet emission region was imaged using a Chronos 2.1-HD high-speed video camera with a Nikkor macro lens (60 mm, 1:2.8) under bright field illumination to verify the cone-jet mode in all experiments.

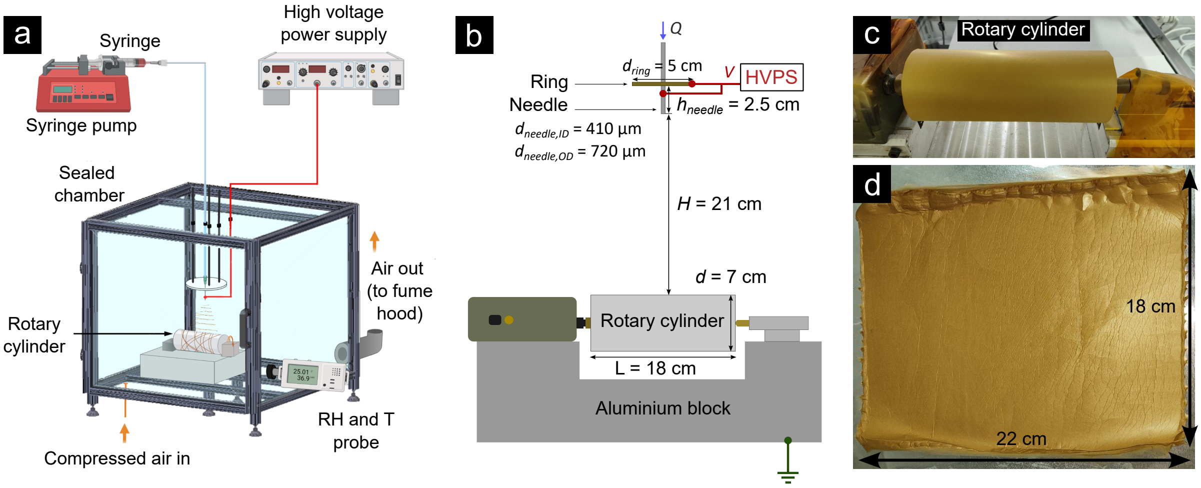

Figure S1. (a) Electrospinning chamber setup (designed by iCAD Assembler and BioRender. DewLab, G. (2026) `https://BioRender.com/dufy89e`). (b) Front-view schematic of the collection setup (not drawn to scale). (c) Rotary cylinder after fiber collection. (d) Lignin-based electrospun mat on top of the unrolled aluminum foil substrate.

To prevent the presence of bubbles, the lignin/PEO solution was poured into the syringe barrel to the brim. Any air remaining inside the syringe was then removed, and the plunger was inserted. The syringe was then mounted on the syringe pump. The solution was pushed into the feed line until it dripped from the end of the needle tip. Immediately thereafter, high voltage was applied to maintain the polymer ejection over time.

Jet rotational motion

When using a photo camera, because of the low screen refresh rate, rotational motion was registered as a double image of the cone on the camera screen and the photo shots, creating the illusion of two cones forming either simultaneously or intermittently, and at irregular positions in the field of view. To better resolve the events in time, a high-speed video camera was incorporated into the setup. The observed imaging revealed that both the cone and the emitted jet underwent a perfectly continuous and stable rotational motion around the needle axis. This rotational behavior was consistently observed in almost all solutions that could be electrospun (Figure S2).

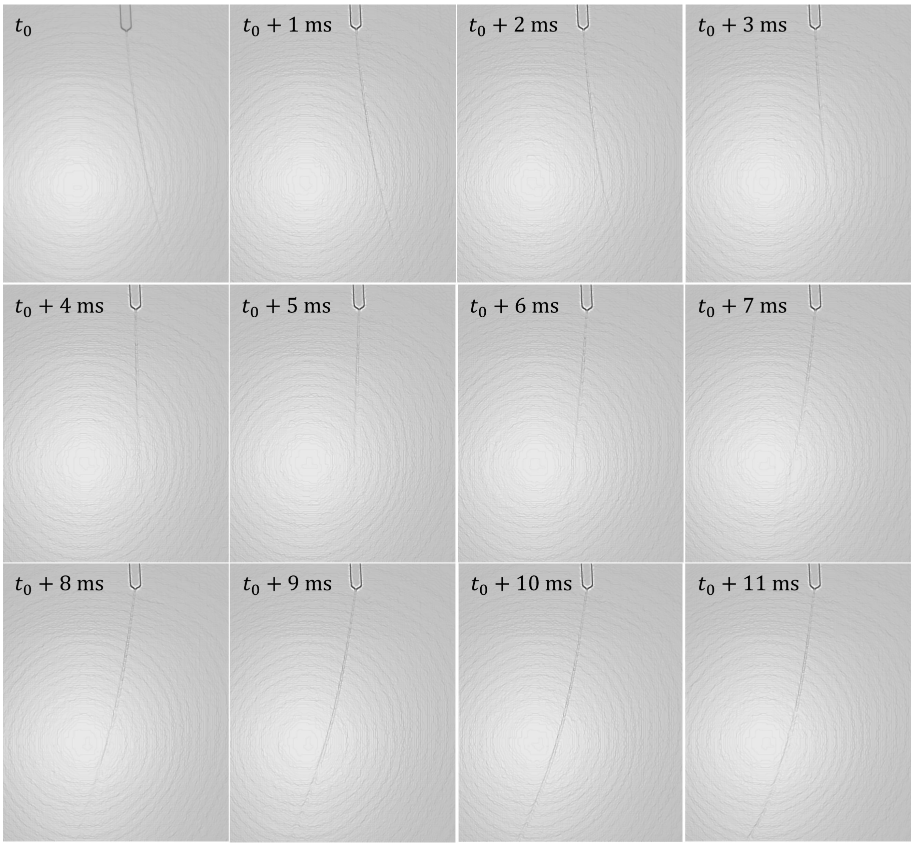

Figure S2. Time sequence of the Taylor cone and jet during electrospinning of SOL/PEO-600 (90/10 w/w) 10 wt % in 0.5 M NaOH. Operational parameters: Q = 0.5 mL/h, V = 12.5 kV., H = 21 cm, RH = 38.6–32.4, T = 22.9–23.2°C. Recorded with a Chronos 2.1-HD high speed camera with a Nikkor macro lens (60 mm, 1:2.8) and edited using IrfanView v 4.70. The OD of the needle equals 720 μm. Image processed using IrfanView v4.70 and PowerPoint to adjust brightness/contrast, color inversion, and border highlighting to increase the visibility of the jet motion.

Ternary plot interpretation aid

The solution compositions that were tested for electrospinning are represented in a composition ternary diagram to identify the regions of stable electrospinnability. To aid in the interpretation of these compositional data, a reference key is provided in Figure S3. This scheme allows for a clear visualization of the concentration of each component in the solution for a given point on the graph, as shown. The concentrations are indicated by the red arrows that point toward the three composition axes. Each arrow runs parallel to one side of the diagram. In addition, the total polymer concentration (lignin plus binder polymer) is easily obtained by running a diagonal line, parallel to the binder polymer axis, as shown. Its intersection with the Lignin axis indicates the total polymer concentration. Finally, the lines of constant lignin/binder polymer ratio are indicated by straight dotted lines like the one shown.

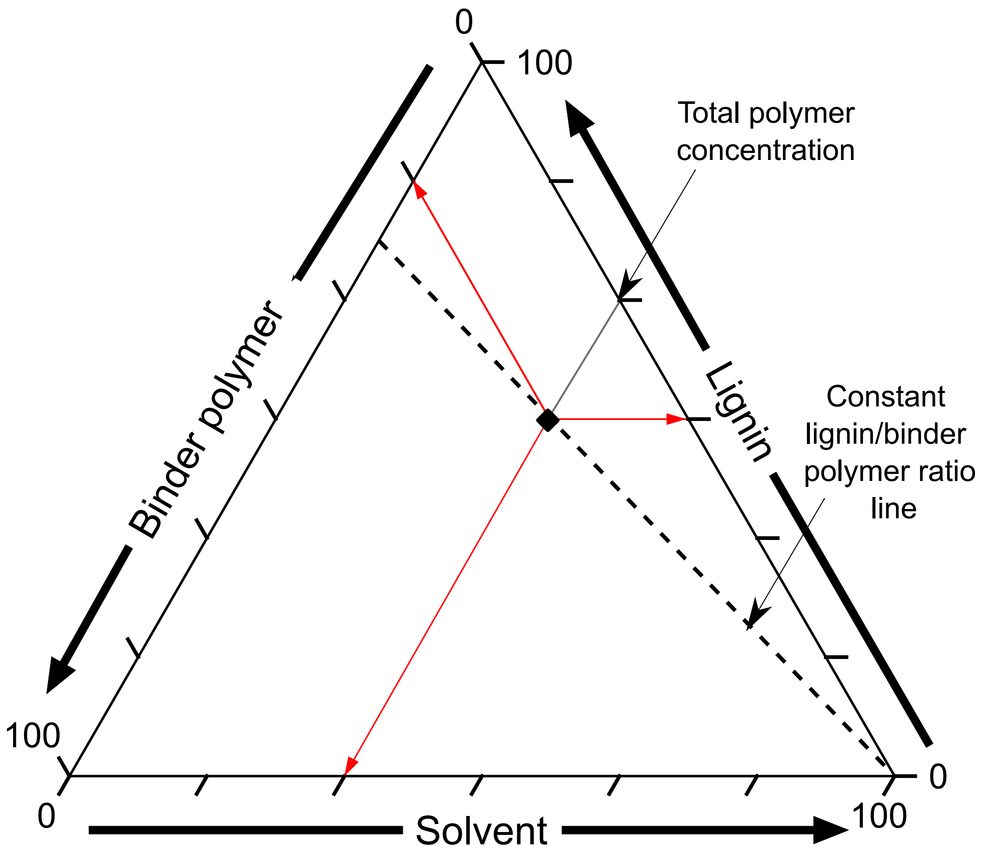

Figure S3. Reference key for interpreting the compositional data of the ternary diagrams presented in this work. The graph considers a data point (indicated by the filled diamond) and its compositional data include the concentrations of each component (indicated by the red arrows) and the total polymer concentration. In addition, the point belongs to a line of constant lignin/binder polymer ratio, as shown.

Entanglement number

The concept of polymer-chain entanglements has been widely used to establish criteria for electrospinnability of a solution.[2,3] The average number of entanglements per chain or n_e can be easily calculated as a function of the entanglement molecular weight in solution $(M_e)_{soln}$, the weight-average molecular weight M_w, and the polymer volume fraction ϕ_p. Therefore, its use has gained wide acceptance in the electrospinning community.

$$n_e = \frac{M_w}{(M_e)_{soln}} = \frac{\phi_p M_w}{M_e} \tag{1}$$

For a single polymer solution, n_e is used as a predictor of the obtained morphology: beads ($n_e < 2$), beads with incipient fibers ($n_e \sim 2$), beaded fibers ($n_e > 1$), or fibers ($n_e > 3.5$). These critical values are valid for the simple case of the good solvent with non-specific polymer–polymer interaction.[2] Here, we have a more complicated situation since the binder polymer and lignin molecules have a specific chemical interaction. Despite this, the spinnability criteria based on

n_e computed solely for the PEO is used as a guide, together with other inputs, to interpret the observed trends in the stable compositions.

Table S2 shows the predicted fiber morphology of PEO fibers depending on the PEO concentration, computed with Equation 1 with parameters ρ_{PEO} is 1.13 g/cm^3, $\rho_{solvent}$ is 1.02 g/cm^3, and M_e is 2 kDa.[4] We have taken M_w as 600 kDa although this is the viscosity-average molecular weight, or M_ν. This is acceptable except for broad polymer weight distributions.[5] Therefore, strictly speaking, since M_w is greater than M_ν, the concentrations at the transition entanglement numbers in the table represent upper limits.

Table S2. PEO concentrations estimated using Eq. 1 for PEO-600 depending on the entanglement numbers (n_e) in each regime and regime transition as the concentration is raised.

n_e	ϕ_p ($\times 10^{-3}$)	PEO (wt %)	Transition	Expected morphology
< 2.0	< 6.8	< 0.75	–	Beads
~ 2.0	6.8	0.75	Fiber initiation	Beads with incipient fibers
2.0–3.5	$6.8 < \phi_p < 12$	0.75 < wt % < 1.30	–	Beaded fibers
~ 3.5	1.2	1.30	Complete fiber formation	Fibers

Rheological characterization

The Cross model was applied to describe the shear-rate dependence of viscosity, allowing the estimation of a characteristic time constant associated with the transition from Newtonian to shear-thinning regimes.[6] The Cross model is described in Equation 2

$$\frac{\eta - \eta_\infty}{\eta_0 - \eta_\infty} = \frac{1}{1 + (\lambda \dot{\gamma})^n} \tag{2}$$

where λ represents an empirical characteristic time for the onset of shear-thinning used to estimate the longest relaxation time, η_0 is the zero-shear viscosity, η_∞ is the infinite-shear viscosity and n is a parameter that characterizes the strength of shear-thinning. Although λ is sometimes used as an approximate indicator of the longest relaxation time, it should not be interpreted as a true viscoelastic relaxation time, since the Cross model describes purely viscous (steady state) behavior rather than viscoelastic dynamics. The Cross model was used solely to describe the shear-thinning behavior of the solutions (steady state viscous response), not their viscoelastic properties.

The average values of these parameters (from three independent measurements on each sample) are presented in Table S3. As the lignin/PEO ratio increases, the zero-shear viscosity of the solutions η_0 decreases markedly (from 1874 mPa·s to 1.4 mPa·s for SOL and from 862 mPa·s

to 1.4 mPa·s for HOL). A decreasing trend is also observed with the η_∞ and λ. Meanwhile, n increases with the lignin/PEO ratio, suggesting higher shear-thinning effect as the polymer entanglements becomes smaller.

Table S3. Cross model constants of OL/PEO-600 solutions in 0.5 M NaOH.

Lignin	Lignin/PEO weight ratio	Poly. (wt %)	OL (wt %)	PEO (wt %)	η_0 (mPa·s)	η_∞ (mPa·s)	λ (ms)	n
-	0/100	2.5	0.0	2.5	105 ± 3	12 ± 13	0.8 ± 0.5	0.74 ± 0.13
SOL	50/50	8.1	4.1	4.0	1,874 ± 54	96 ± 10	14.6 ± 0.5	0.687 ± 0.014
		10.0	5.0	5.0	5,768 ± 407	87 ± 10	34 ± 3	0.656 ± 0.005
	75/25	10.1	7.5	2.6	834 ± 39	109 ± 2	8.9 ± 0.3	0.768 ± 0.011
	90/10	10.0	9.0	1.01	82 ± 4	15 ± 3	1.4 ± 0.4	–
		12.1	10.8	1.23	299 ± 6	66 ± 1	6.02 ± 0.04	0.761 ± 0.012
		15.0	13.5	1.5	2,522 ± 304	127 ± 30	34 ± 3	0.56 ± 0.03
	92/8	14.4	13.2	1.19	447 ± 23	77 ± 1	7.8 ± 0.3	0.72 ± 0.01
	96/4	10.0	9.6	0.46	17.8 ± 0.5[a]	–	–	–
		14.0	13.4	0.57	147 ± 36	45 ± 8	3.5 ± 0.8	0.87 ± 0.22
	98/2	14.5	14.2	0.30	26.7 ± 0.2	20.6 ± 0.2	7.43 ± 0.04	–
	100/0	7.5	7.5	0.00	1.44 ± 0.07[a]	–	–	–
HOL	50/50	8.08	4.06	4.02	862 ± 29	94 ± 7	6.3 ± 0.2	0.80 ± 0.02
		10.0	5.0	5.0	2,210 ± 290	107 ± 5	11 ± 2	0.74 ± 0.01
	75/25	10.0	7.5	2.5	218 ± 20	58 ± 10	2.3 ± 0.3	0.90 ± 0.09
	90/10	10.0	9.0	1.04	34 ± 5[a]	–	–	–
		12.0	10.8	1.22	66 ± 1	28.7 ± 0.9	1.3 ± 0.1	0.93 ± 0.03
		15.0	13.5	1.5	375 ± 3	96 ± 11	3.4 ± 0.5	0.80 ± 0.05
	92/8	14.3	13.2	1.15	105.3 ± 0.4	48 ± 1	2.3 ± 0.1	–
	96/4	10.1	9.6	0.44	7.4 ± 0.0[a]	–	–	–
		14.0	13.4	0.58	56 ± 10[a]	–	–	–
	98/2	14.5	14.2	0.30	8.6 ± 0.5[a]	–	–	–
	100/0	7.5	7.5	0.00	1.36 ± 0.04[a]	–	–	–

[a] Newtonian behavior. Cross model is not applicable. [b] Note that λ corresponds to the characteristic time from the Cross model, empirically related to the onset of shear-thinning.

Overall, softwood organosolv lignin combined with PEO exhibited higher viscosity than its hardwood counterpart.

The specific viscosity data have been fitted to $\eta_{sp} = A\ [Polymer]^B$, where $[Polymer]$ stands for the total polymer concentration, and A and B are linear functions of the polymer ratio x =[PEO]/[OL]: $A \equiv A_1 + A_2 x$ and $B \equiv B_1 + B_2 x$. The compositions considered in the fitting are those within the region defined by [OL]/[PEO] $\geq$ 90/10 and polymer concentration between 10 and 14.5 wt % for which electrospinning was possible (resulting in either fibers or beaded fibers, as indicated in Table S1, see also Figure 2), plus the 100/0 composition (Table S3). The model parameters were estimated by weighted least-squares fitting in logarithmic space, minimizing the

sum of squared logarithmic residuals normalized by the relative standard deviation in the specific viscosity $\sigma(\eta_0)/\eta_{sp}$. The parameters A and B are reported in Table S4.

Table S4. Parameters A and B for the fitting $\eta_{sp} = A\ [Polymer]^B$ at various OL/PEO weight ratios, applicable to electrospinnable compositions within the region defined by [OL]/[PEO] $\geq$ 90/10 and 10 $\leq$ $[Polymer] \leq 14.5$ wt % ($[Polymer] \equiv$ [PEO]+[OL]).

Lignin	Lignin/PEO weight ratio	A	B
SOL	90/10	$6.78 \cdot 10^{-5}$	6.13
	92/8	$1.45 \cdot 10^{-4}$	5.61
	96/4	$2.89 \cdot 10^{-4}$	4.63
	98/2	$3.57 \cdot 10^{-4}$	4.17
HOL	90/10	$4.69 \cdot 10^{-7}$	7.55
	92/8	$5.35 \cdot 10^{-7}$	7.17
	96/4	$6.59 \cdot 10^{-7}$	6.45
	98/2	$7.18 \cdot 10^{-7}$	6.12

The crossover relaxation time (τ_c) was determined from small Amplitude Oscillatory Shear (SAOS) tests *via* small amplitude (1 %) within the linear viscoelastic region (1–20 Hz), as the inverse of the crossover frequency (ω_c) at which the storage and loss moduli ($G'(\omega)$ and $G''(\omega)$) intersect, i.e.,

$$G'(\omega_c) = G''(\omega_c) \quad \text{and} \quad \tau_c = \frac{1}{\omega_c} \tag{3}$$

This parameter represents the characteristic relaxation time that separates the predominantly elastic (solid-like) and viscous (liquid-like) regimes of the solution.

Fiber diameter data

Table S5. Fiber diameter data for the electrospinnable compositions. N is the sample size for the fiber diameter (reported as mean ± standard deviation). "Polymer wt %" refers to the percentage of total polymer weight of PEO and lignin in the solution.

Lignin	PEO M_ν (kDa)	Lignin/PEO weight ratio	Polymer wt %	Fiber diameter (nm)	N
SOL	600	50/50	8	541 ± 90	22
			10	644 ± 98	40
		75/25	10	825 ± 54	16
		90/10	10	634 ± 50	38
			12	765 ± 56	220
		96/4	10	318 ± 50	54
			14	798 ± 93	17
	1,000	98/2	14.5	1,380 ± 370	14
	5,000	99/1	15.3	2,500 ± 520	71
HOL	600	50/50	8	597 ± 89	24
			10	487 ± 71	72
		75/25	10	395 ± 51	24
		90/10	10	299 ± 42	65
			12	558 ± 64	28
			15	609 ± 99	84
		96/4	14	460 ± 72	34
	1,000	98/2	14.5	550 ± 110	23

Additional fiber morphologies

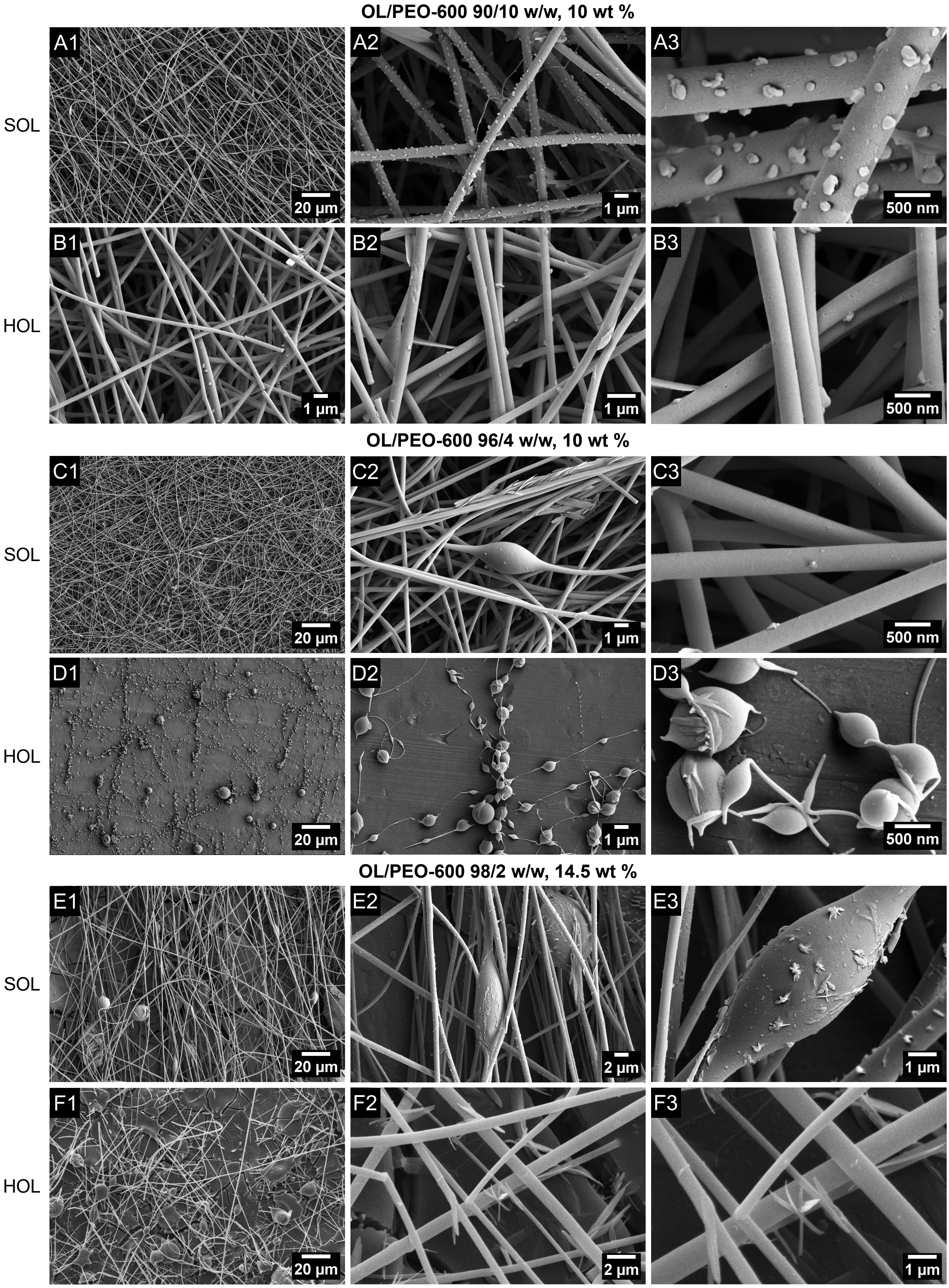

Figure S4. SEM images of fibers electrospun from OL/PEO-600 (A,B) 90/10, (C,D) 96/4 (E,F) 98/2 weight ratios and total polymer concentration of (A-D) 10 wt % and (E,F) 14.5 wt %.

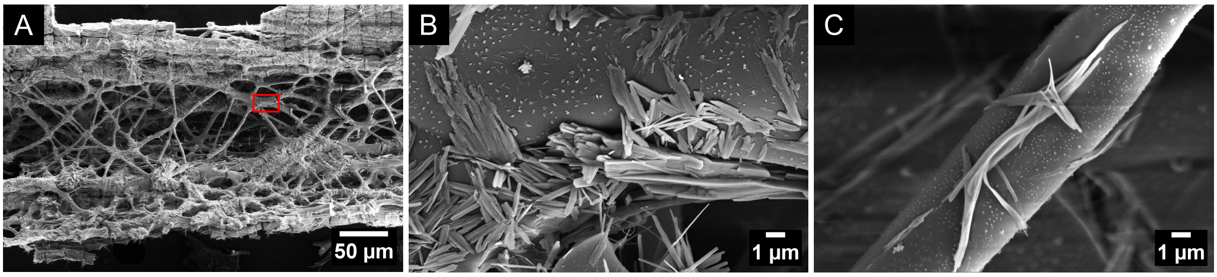

Figure S5. FESEM images of fibers electrospun from SOL/PEO-600 90/10 weight ratio and total polymer concentration of 15 wt %: (A) low-magnification, (B) high-magnification of panel A (red square), and (C) high-magnification (fiber).

Additional electrospun fibers for PEO-5000 are presented in Figure S6:

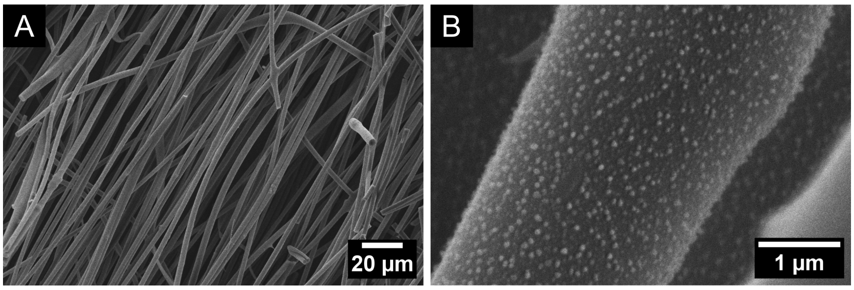

Figure S6. SEM images of fibers electrospun from SOL/PEO-5000 99/1 weight ratios and total polymer concentration of 15.3 wt %: (A) low-magnification and (B) high-magnification.

Reproducibility tests of electrospun nanofibers

To evaluate the reproducibility of the samples and the effect of storage, two experiments were conducted using a solution of SOL/PEO-600 90/10 (w/w).

In the first experiment, to measure the effect of humidity during storage, samples from the same batch were imaged after being stored for 15 days under two different conditions: dried conditions in a desiccator (Figure S7(a-c) and under ambient conditions at RH∼40% (Figure S7(d). Additionally, to confirm the homogeneity of fiber size distribution, a histogram of the fiber widths (765 nm ± 56 nm) for all samples with both conditions is shown in Figure S7 (e).

There were no visible differences in fiber morphology and diameter distribution between different regions of the mat: left side (782 nm ± 50 nm), center (749 nm ± 46 nm), and right side (755 nm ± 67 nm). Similarly, no morphological variations were observed under different storage conditions, exhibiting an average diameter width of 776 nm ± 53 nm under ambient conditions. This idea was confirmed by histogram analysis (Figure S7(e)), which exhibited consistent a standard deviation of 56 nm, thereby indicating that the electrospun fibers were uniform.

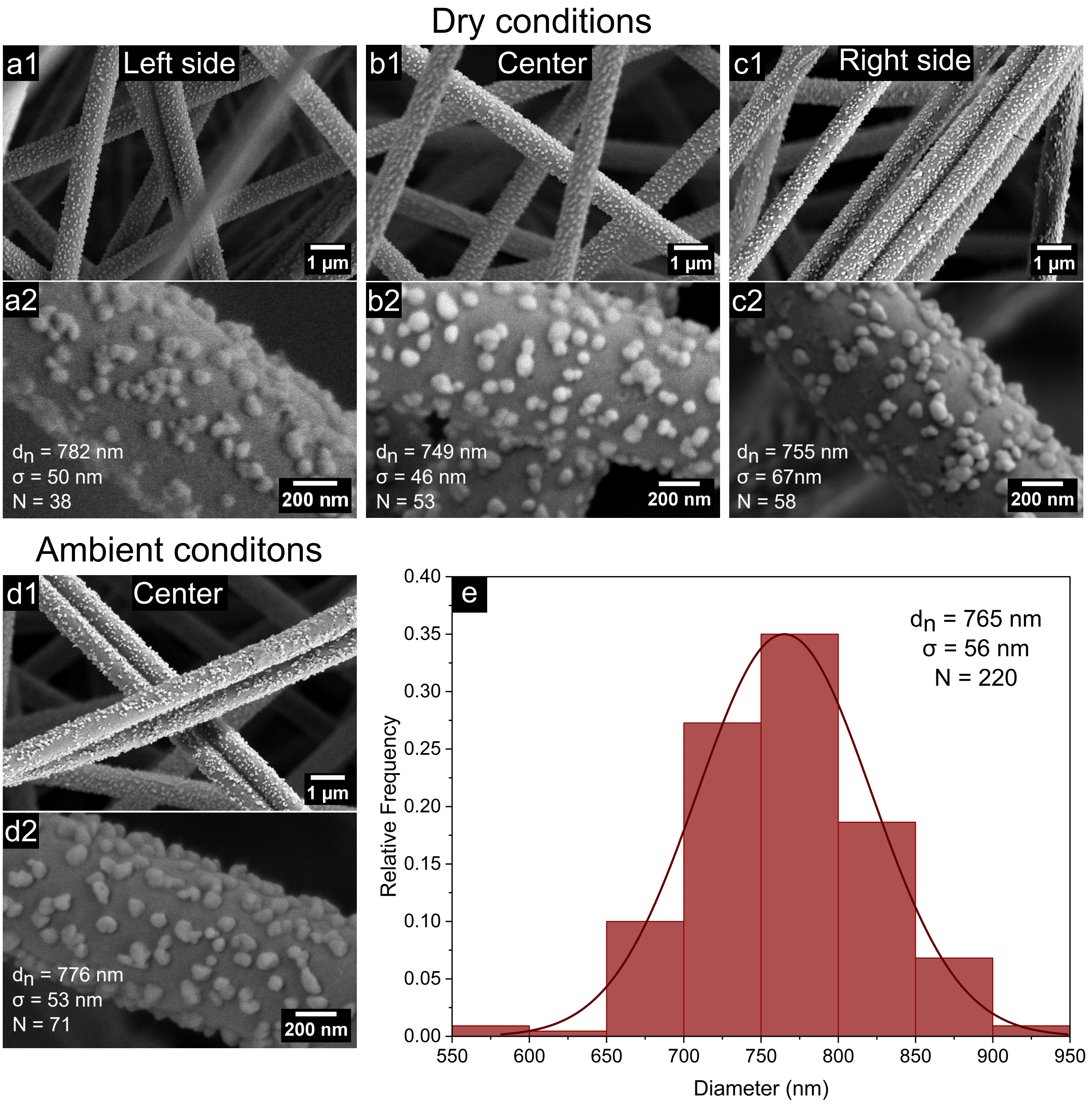

Figure S7. FESEM images of electrospun lignin-based fibers stored under dried the dessicator (a-c) and under laboratory conditions (d). Solution of SOL/PEO-600 90/10 w/w 12 wt % total polymer solution in 0.5 M NaOH was used. (e) Fiber-diameter based on FESEM images. d_n: average fiber diameter, σ: standard deviation, N: number of analyzed fibers.

In a second experiment, the same solution composition as in Figure S7 was prepared and electrospun. Samples were evaluated after 1 day of storage in the desiccator (Figure S8). This experiment was designed to demonstrate that there is no variation in the morphology of the electrospun fibers from SOL/PEO-600 90/10 (w/w) solution.

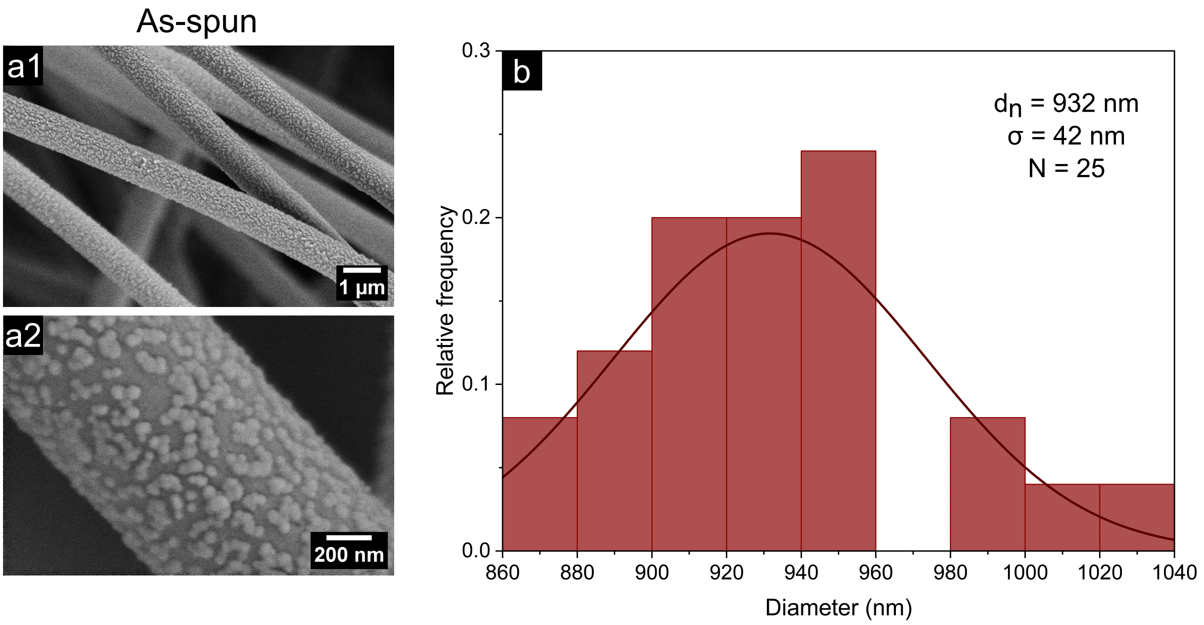

Figure S8. FESEM images of as-spun lignin-based fibers (a) from solution of SOL/PEO-600 90/10 w/w 12 wt % total polymer solution in 0.5 M NaOH was used. (b) Fiber-diameter based on FESEM images. d_n: average fiber diameter, σ: standard deviation, N: number of analyzed fibers. Samples was stored in the desiccator for 1 day.

The electrospun fibers of Figure S7 exhibited a narrow size distribution with a submicron mean diameter and a standard deviation of 765 nm ± 56 nm (sample size N of 220). In the second electrospun fibers, we can see that the fibers are slightly thicker, with a mean diameter of 932 nm ± 42 nm (sample size N of 25). Although the first electrospun fibers were thinner, fibers as-spun exhibited a similar appearance, showing Na_2CO_3 particles on the surface.

Additional fiber cross sections by FIB-FESEM

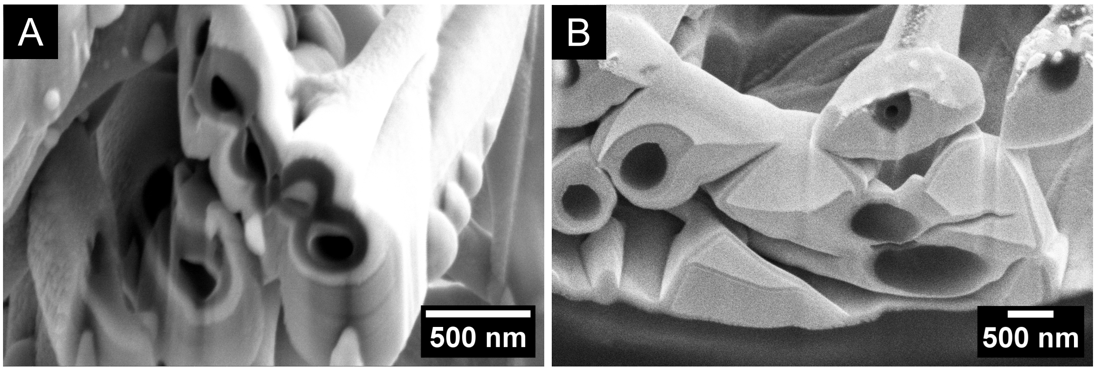

Figure S9. FIB-FESEM cross sections of HOL fibers electrospun from feed solutions with 90/10 lignin/PEO-600 weight ratios and total polymer concentrations of: (A) 10 wt %, and (B)15 wt %.

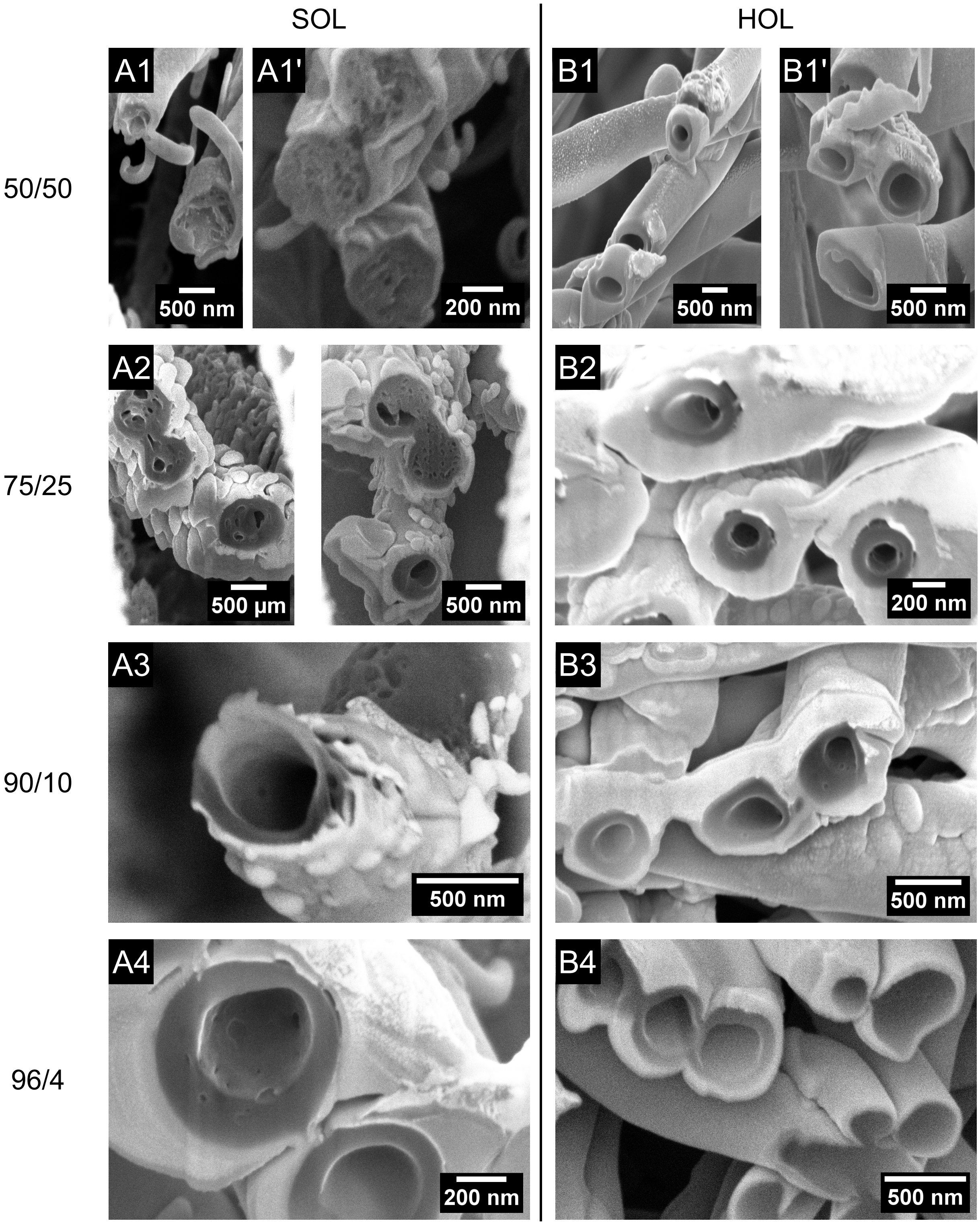

Figure S10. FIB-FESEM cross sections of SOL (A panels) and HOL (B panels) fibers electrospun from feed solutions with different lignin/PEO-600 weight ratios and total polymer concentrations of: (1) 50/50 (w/w), 8 wt %; (2) 75/25 (w/w), 10 wt %; (3) 90/10 (w/w), 12 wt %; (4) 96/4 (w/w), 14 wt %. Same compositions as used in Figure 5.

In the study on the internal structure of the fibers, many fibers were milled and viewed by FIB-FESEM to determine (with enough statistical power) whether the electrospun nanofibers

were totally compact or hollow, or had a mixed situation with voids. Figure S9 and S10 contain additional images to the ones shown in the main article.

Surface characterization

The fiber surface is presented with high magnification in Figure S11, with small pores on the surface, which are visible in panel c. As described in the article, the external structures of the fibers vary greatly in shape depending on composition. A clear example of this variation is panel a and b, which exhibited acicular (crystals) and granular structures, respectively.

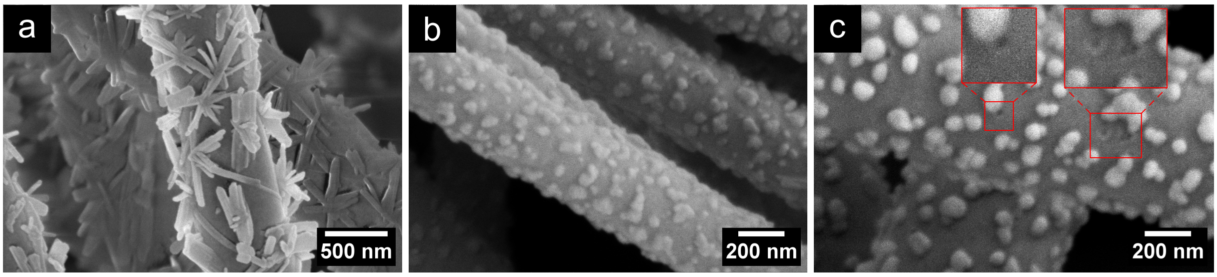

Figure S11. High magnification FESEM top views of SOL (a,c) and HOL (b) fibers electrospun from feed solutions with lignin/PEO-600 weight ratios and total polymer concentrations of: (a,b) 75/25 (w/w), 10 wt %; (c) 90/10 (w/w), 12 wt %.

EDX analysis of the external morphology

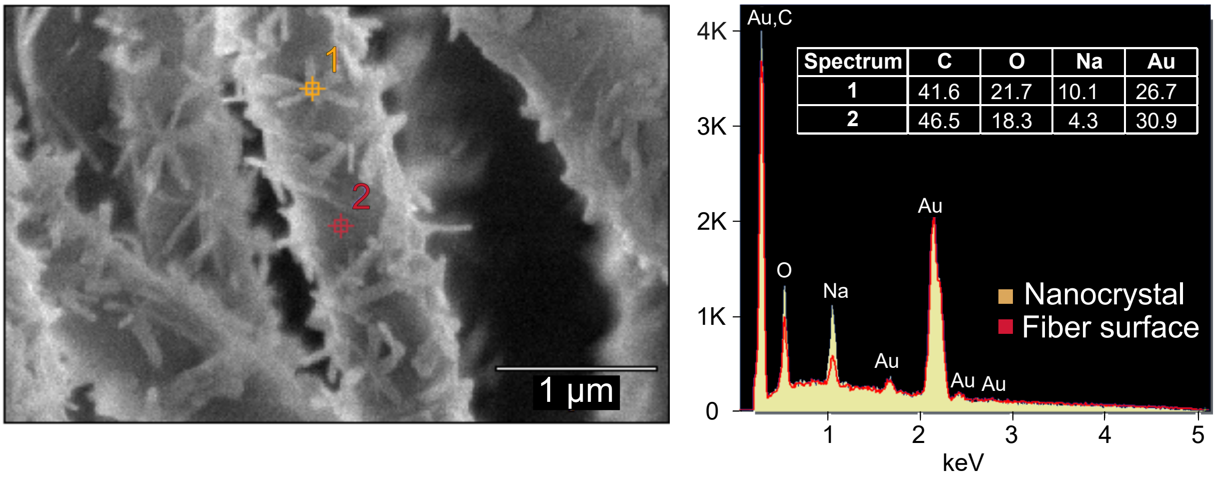

Figure S12. EDX for two positions on an electrospun fiber from the solution of SOL/PEO-600 75/25 at 10 wt % total polymer in 0.5 M NaOH. The atomic percentages (AP) are presented. The AP for gold is attributed to the sputtered coating of this material.

Water evaporation rate estimates

The solvent evaporation rates from the Taylor cone and from the jet are first estimated by models that assume that they are small; then this initial assumption is confirmed. Consistently, we also assume the liquid to be at ambient temperature and the interfacial composition to be 100% water. Both assumptions are conservative, defining a worst-case scenario. We further assume a diffusion-limited evaporation mechanism.

For the Taylor cone, we estimate the solvent evaporation rate as one-half of that from a sphere. The differences in shape are glossed over as we only seek an estimate. The vaporization flux (mass evaporated per unit area and per unit time) for a water sphere scales approximately as $J_{vc} \simeq D\Delta c/a$ where D is the diffusion coefficient of water vapor in air, a is the sphere radius, and Δc is the difference in vapor concentration between the surface c_v^{sat} and the surroundings c_v^{∞}; that is, $\Delta c = c_v^{sat} - c_v^{\infty} = c_v^{sat}(1 - RH)$. The rate of evaporation from the cone is $\dot{m}_{vc} = J_{vc}A$, where A is half the sphere surface area, $A = 2\pi a^2$. The rate of water evaporation from half the sphere becomes $\dot{m}_{vc} = J_{vc}A \simeq 2\pi a D c_v^{sat}(1 - RH)$. This quantity must be compared to the solution flow rate pumped into the Taylor cone, or ρQ. The importance of vaporization of water at the Taylor cone is thus governed by the dimensionless ratio $\dot{m}_{vc}/(\rho Q)$. Now, taking a as half the OD of our electrospinning needle ($a = 360$ μm), $D = 0.24\ cm^2/s$ (1 bar, 20°C), $c_v^{sat} = 23\ g/m^3$ (25°C),[7] $RH = 0.3$, $Q = 0.5$ ml/h, and $\rho = 10^3$ kg/m^3, we get that $\dot{m}_{vc}/(\rho Q) = 0.0063$. Since this is a very small quantity, we can neglect evaporation from the Taylor cone.

For the jet, the argument goes similarly. We assume that the jet is slender and that the evaporation rate changes slowly along its length. The local evaporation rate for a section of jet of length l much longer than the jet radius a_j ($l \gg a_j$) is $\dot{m}_{vj} = J_v A_j$ where $J_v \simeq D c_v^{sat}(1 - RH)/a_j$ and $A_j \simeq 2\pi a_j l$. Therefore, $\dot{m}_{vj} \simeq 2\pi l D c_v^{sat}(1 - RH)$, independent of the jet radius and linear in l (a relatively slow dependence, consistent with the above assumptions). The relative importance of water vaporization from the jet is governed by the dimensionless ratio $\dot{m}_{vj}/(\rho Q)$, which for $l = 1$ mm equals 0.017. Thus, after 1 cm of jet length, about 17% of the initial flow has left as water vapor from the jet. In conclusion, water evaporation is small during the jet ejection but should have become non-negligible after a jet length of several centimeters. Note that, as the evaporation becomes important, jet cooling will take place, slowing the evaporation down.

References

[1] Kiselev, P.; Rosell-Llompart, J. Highly aligned electrospun nanofibers by elimination of the whipping motion. *Journal of Applied Polymer Science* **2012**, *125*, 2433–2441.

[2] Shenoy, S. L.; Bates, W. D.; Frisch, H. L.; Wnek, G. E. Role of chain entanglements on fiber formation during electrospinning of polymer solutions: good solvent, non-specific polymer–polymer interaction limit. *Polymer* **2005**, *46*, 3372–3384.

[3] Bodnár, E.; Grifoll, J.; Rosell-Llompart, J. Polymer solution electrospraying: A tool for engineering particles and films with controlled morphology. *Journal of Aerosol Science* **2018**, *125*, 93–118.

[4] Rubinstein, M.; Colby, R. H. *Polymer Physics*; Oxford University Press, 2003; Chapter 9 - Entangled polymer dynamics, pp 361–422.

[5] Graessley, W. W. *Polymeric Liquids & Networks: structure and properties*; Garland Science, 2004; Chapter 5 - Dilute Solution Characterization, pp 167–202.

[6] Barnes, H.; Hutton, J.; Walters, K. *An Introduction to Rheology*; Rheology series; Elsevier Science & Technology, 1989.

[7] William M. Haynes, Ed. *CRC Handbook of Chemistry and Physics*, 97th ed.; CRC Press, 2016; Chapter 6 - Fluid Properties, p 2670.
